# Protecting playgrounds: local-scale reduction of airborne particulate matter concentrations through particulate deposition on roadside ‘tredges’ (green infrastructure)

**DOI:** 10.1038/s41598-022-18509-w

**Published:** 2022-08-20

**Authors:** Barbara A. Maher, Tomasz Gonet, Vassil V. Karloukovski, Huixia Wang, Thomas J. Bannan

**Affiliations:** 1 Centre for Environmental Magnetism and Palaeomagnetism, Lancaster Environment Centre, Lancaster University, Lancaster, LA1 4YQ UK; 2 School of Environmental and Municipal Engineering, Xi’an University of Architecture and Technology, Xi’an, 710055 Shaanxi People’s Republic of China; 3 School of Earth, Environmental and Atmospheric Science, University of Manchester, Manchester, UK; 4 Present Address: Jaguar Land Rover, Gaydon, Lighthorne Heath, Warwick, CV35 0BJ UK

**Keywords:** Earth and environmental sciences, Environmental sciences

## Abstract

Exposure to traffic-related particulate air pollution has been linked with excess risks for a range of cardiovascular, respiratory and neurological health outcomes; risks likely to be exacerbated in young children attending schools adjacent to highly-trafficked roads. One immediate way of reducing airborne PM concentrations at the local (i.e., near-road community) scale is installation of roadside vegetation as a means of passive pollution abatement. Roadside vegetation can decrease airborne PM concentrations, through PM deposition on leaves, but can also increase them, by impeding airflow and PM dispersion. Critical to optimizing PM removal is selection of species with high particle deposition velocity (*Vd*) values, currently under-parameterised in most modelling studies. Here, the measured amounts of leaf-deposited magnetic PM after roadside greening (‘tredge’) installation, and measured reductions in playground PM, particle number and black carbon concentrations demonstrate that air quality improvements by deposition can be achieved at the local, near-road, community/playground scale. PM deposition on the western red cedar tredge removed ~ 49% of BC, and ~ 46% and 26% of the traffic-sourced PM_2.5_ and PM_1_, respectively. These findings demonstrate that roadside vegetation can be designed, installed and maintained to achieve rapid, significant, cost-effective improvement of air quality by optimising PM deposition on plant leaves.

## Introduction

Exposure to the traffic-related air pollution (TRAP) associated with proximity to major roads has been linked with excess risks for a range of cardiovascular and respiratory health outcomes. Air pollution comprises a mix of gases and solid particles of varying sizes. Exposure to fine particulate matter (PM_2.5_, < 2.5 μm in aerodynamic diameter) in air pollution is reportedly the largest environmental risk factor contributing to cardiovascular mortality and morbidity, globally^1–3^, and causes about six to nine million premature deaths each year^4,5^.

Evidence increasingly shows that exposure to airborne PM is also linked with impaired neuro-development and cognitive function. Robust correlations have been found between outdoor PM concentrations and reduced brain function, in different geographic settings and age groups. Epidemiological studies have shown measurable neurological impacts at PM concentrations below current policy limits/recommendations (e.g.^6,7^).

Infants and young children are particularly vulnerable to the impacts of air pollution, incurring health burdens blighting their entire life span. Their major organs are still undergoing development. They inhale higher PM doses given their lower lung volume and higher breathing rate (double that of an adult per unit of body weight)^8^. As pedestrians, they are closer in height to peak concentration height (~ 0.3 m) of vehicle-derived PM^9^. While residential proximity to major roads has been associated with adverse health outcomes (e.g.^10,11^), school location may also be an important determinant of children's exposure to traffic-related pollutants^12,13^. Internationally, millions of children are exposed to elevated TRAP levels by attending schools located close to major roads, especially those attending schools in low-income neighbourhoods (e.g.^14–16^). Children attending schools in areas with high levels of particulate air pollution displayed deficits in cognitive development in Barcelona, Spain^17,18^. Other epidemiological studies indicate cognitive deficits in young people exposed to high TRAP concentrations (e.g.^19,20^). Long-term PM_2.5_ exposure has been linked with behavioural problems; e.g., increased delinquency of urban-dwelling adolescents in Southern California, USA^21^. Airborne PM is also linked with incidence of premature birth and low birth weight^22,23^, and mortality in paediatric, adolescent and young adult patients with specific cancers^24^. Exposure to carbonaceous TRAP is associated with increased all-cause, neurodegenerative and cardiovascular mortality risks (^25–27^).

Globally, road traffic constitutes the largest (albeit declining^28^) single source of outdoor air pollution in urban environments^29^. Vehicles and associated road wear produce airborne PM very effectively, from a range of sources, and with a range of particle sizes and compositions. Gases, such as nitrogen dioxide, are emitted from exhausts, as are semi-volatile and solid particles, often a mixture of carbon and various bioreactive metal species (including iron, manganese, nickel, chromium, lead, platinum). Solid particles are produced abundantly from wear of brakes, tyres, and road surfaces^30^ as well as exhausts. Those produced by brake wear are often ultrafine in size (< 100 nanometers, nm) and rich in iron, including metallic and magnetic forms of iron, such as magnetite^31–33^. Hence, magnetic measurements of airborne PM often demonstrate strong direct correlation with more conventional measurements of PM and NOx, for example^34–36^.

The causal role of airborne PM in driving adverse health outcomes in children is attested independently by observed mitigation of health impacts after reducing outdoor PM concentrations. Critically, even small improvements in air quality result in measurably improved health outcomes. In East Germany, the decline of coal combustion-related air pollution after reunification was associated with improved lung function in children^37^. Within a cohort of children in California, those who moved to areas with lower levels of PM_10_ (< 10 µm aerodynamic diameter) showed increased lung function growth; those who moved to more polluted areas experienced decreased lung function growth^38^.

Given the health impacts of TRAP, especially for young children, policy-driven reduction of PM emissions is essential and urgent but in practice is slow and incremental. One immediate and practical way of reducing airborne PM concentrations at the local (i.e., near-road community) scale may be the installation of green infrastructure, such as roadside trees/shrubs/hedges/grasses (e.g.^9,39–42^). This role for roadside vegetation as a means of passive pollution abatement (combined with other ecosystem services) is increasingly recognised globally. A fast-growing body of published papers has reported a remarkably large range of experimental (real-world) and modelled outcomes of the effects of roadside greening on PM levels. A recent review by the Nature Conservancy^43^ collates much of this disparate evidence.

Critically, roadside vegetation can decrease airborne PM concentrations, through PM deposition on leaves, but can also lead to increased PM concentrations, by impeding airflow and altering PM dispersion. The ‘competition’ between these two mechanisms determines the air quality at pedestrian/road user level, and the amount of PM leaving the road area/vegetation canopy^44^.

Recent modelling-based papers and expert reviews (e.g.^45^) state that the magnitude of PM *deposition* on urban vegetation is small in most street contexts; ‘dwarfed’^46^ by the effects that street vegetation has on *dispersion* of local PM. Such studies mostly use computational fluid dynamics (CFD) to simulate PM emission, dispersion and deposition, and, typically, indicate small reductions (i.e., a few percent) in PM_10_ or PM_2.5_ concentrations by deposition onto (mostly existing, often very tall) roadside vegetation (e.g.^47–50^). Common to most of these studies is their standardised parameterisation of a critical factor, the velocity of particle deposition to leaf surfaces.

The deposition velocity (*V*_*d*_) is the quotient of the particle mass flow rate (F_p_, in µg m^−2^ s^−1^) towards the leaf surface and the atmospheric particle concentration (C_p_, in µg m^−3^), normally given in m s^−1^ or cm s^−1^:

$$V_{d} = {\text{ F}}_{{\text{p}}} /{\text{C}}_{{\text{p}}}$$

*V*_*d*_ varies with many factors, including particle size and density, surface morphology, meteorological conditions (wind speed, humidity, rainfall, atmospheric stability), ambient concentrations of other (e.g. gaseous/semi-volatile) pollutants. Such multifactorial, often stochastic variability in *V*_*d*_ may account for the diverse reported PM removal rates by vegetation. However, *V*_*d*_ also varies markedly with plant species (and sub-species/cultivar), via leaf surface traits, leaf size, morphology and number, canopy structure (e.g.^35,41,51–55^), and as yet poorly known physicochemical interactions between leaves and airborne particles, and between particles (especially for sub-micrometre PM).

The *modelled* effectiveness of PM removal by vegetation depends substantively on the *V*_*d*_ value selected for the model^41,44,56–58^. The ‘standard’ *V*_*d*_ values used in most modelling studies of PM deposition vs dispersion effects are typically 0.64 cm s^−1^ or 0.1 cm s^−1^ for PM_2.5_ (e.g.^48,49,59^) and 0.2 cm s^−1^ for PM_10_ (e.g.^50^).

Self-evident is that modelled estimates of PM removal by deposition will be low when the ‘standard’ *V*_*d*_ value used in those models is low (i.e., ~ 0.1 to 0.64 cm s^−1^). If the selected *V*_*d*_ is unrealistically low, then such studies may misrepresent the potential efficacy of roadside greening, especially at the local, near-road community/playground scale, and in the context of new roadside greening schemes, where high-*V*_*d*_ species can be selected explicitly (e.g.^41,58^).

The species-dependence of *V*_*d*_ (e.g.^35,60–62^) appears of key and currently overlooked importance in optimising roadside greening. (‘Plant species’ hereafter also refers to sub-species and cultivar). Using magnetic particle loadings as a proxy for PM_10_, Mitchell et al.^35^ reported (magnetic) species-specific *V*_*d*_ values, varying as a function of leaf micro-topography, especially hairiness and rugosity (i.e., small-scale variations in leaf surface height/topography); as subsequently attested, for a diverse species range, by e.g.^63^. Lowest *V*_*d*_ values (0.5 to 0.9 cm s^−1^) were measured for sweet chestnut, elder, elm, and willow (similar, long term average values, 0.7 to 1.07 cm s^−1^, were reported for mixed stands of sweet chestnut, oak, holly, yew and Scots pine^64^). Mitchell et al.^35^ reported intermediate values (~1.3 to 1.9 cm s^−1^) for sycamore, horse chestnut, ash, and maple; and higher *V*_*d*_ values (2.4 to 4.6 cm s^−1^) for lime, beech, and silver birch (particles agglomerating in extremely high numbers for birch within leaf surface micro-furrows, SI Fig. S1). Even higher *V*_*d*_ values (for PM_1_–PM_10_), > 8 cm s^−1^, have been reported for grassland^65^ and Douglas fir^53^, dependent on/increasing with wind speed^52^.

**Figure 1:**
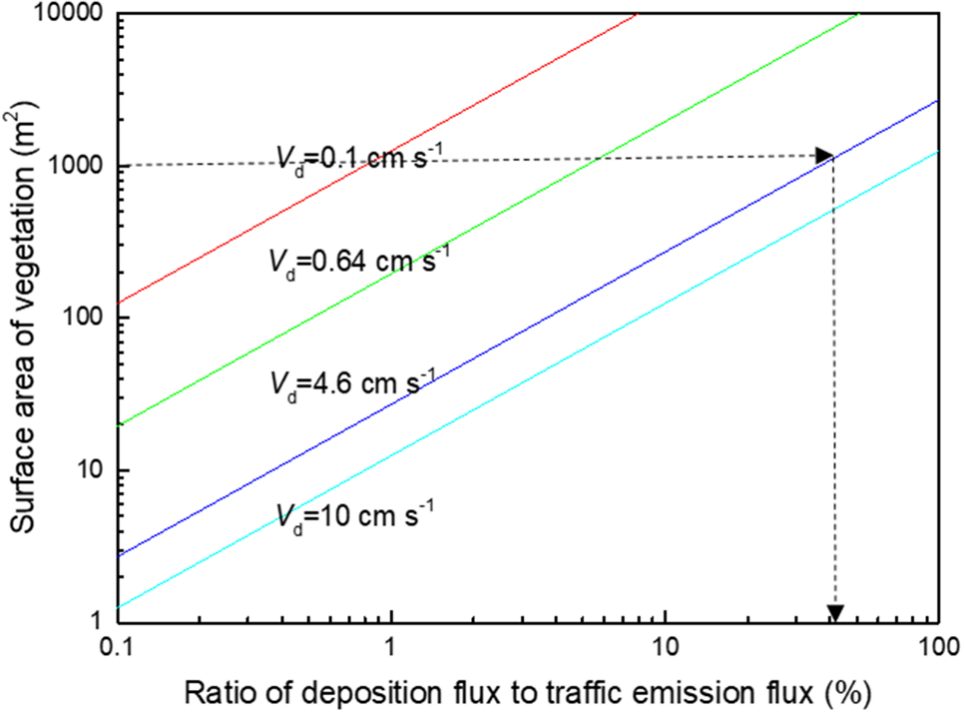
Ratio of vegetation deposition flux to traffic emission flux; depending on surface area of vegetation with differing leaf deposition velocity (*V*_d_) in a street canyon of 100 m length with annual PM_10_ concentration of 37 µg m^−3^, average emission factor 100 µg vehicle^−1^ m^−1^ for PM_10_, traffic intensity of ~ 40,000 vehicles day^−1^ (adapted from^56,58^). The black dashed lines indicate the % PM_10_ removal (~ 40%) by leaf deposition, with 8 silver birch trees (*V*_d_ = 4.6 cm s^−1^, canopy diameter 8 m, total leaf surface area = 125 m^2^/tree).

The significance of leaf *V*_*d*_ parameterisation for estimating depositional removal of local, traffic-derived PM is demonstrated in Fig. 1. Taking, for example, our experimentally-derived *V*_*d*_ of 4.6 cm s^−1^ (silver birch, Mitchell et al., 2010) with leaf surface area of 125 m^2^/tree (canopy diameter 8 m), 8 silver birch trees/100 m street length would remove ~ 40% of the traffic-derived PM_10_ (Fig. 1). Such removal rates appear congruent with indoor PM measurement campaigns for roadside birch^41^ and other green structures (‘hedge’, ‘tree’, species not identified) as summarised by^39,66^.

Conversely, if a conventional, ‘standard’ *V*_*d*_ value of 0.1 or 0.64 cm s^−1^ is assumed (Fig. 1), then the estimated PM removal by those eight trees would be just ~ 1 to 5% of the traffic-emitted PM_10_. Using the low ‘standard’ *V*_*d*_ values, an unfeasibly large number of trees (> 80 trees/100 m road) would be needed, to provide the enormous surface area necessary to compensate for the low assumed *V*_*d*_, and thence increase the estimated PM removal to any significant portion of the local, traffic-emitted PM_10_.

Apparent then from Fig. 1 is that local-scale planting of species selected for their *high V*_*d*_ (i.e., > ~ 2 cm s^−1^), sited close to the local, traffic-related PM sources, could substantively reduce local PM concentrations through leaf deposition. Here, we installed different types of ‘tredges’ (i.e., trees managed as hedges) along the existing wire fences separating three primary school playgrounds from major arterial (non-canyon) roads in Air Quality Management Areas in Manchester, UK, to investigate their efficacy in reducing playground PM, particle number concentrations (PNC) and black carbon (BC).

Air quality and leaf magnetic measurements indicate greatest playground PM reductions (probably by airflow blocking) by the ivy screen (~25% for median PM_2.5_ and PM_1_, 14% for PNC, no reduction in BC), and greatest playground PM reductions (by leaf deposition) by the evergreen western red cedar tredge (~ 23% for median PM_2.5_, 13% for PM_1_, 16% for PNC, 49% for BC). The incidence of short-lived, high-magnitude peaks in PM and PNC was substantially reduced; for example, by 67–82% for PM_2.5_, PM_1_ and PNC behind the western red cedar tredge. These findings highlight that local-scale planting campaigns of high-*V*_*d*_ roadside tredges can contribute immediately to mitigation of health hazard, especially where particularly vulnerable population groups (e.g. young children) are exposed to PM from proximal, heavily-trafficked roads.

## Results

### PM concentrations

Figure 2 shows the PM_10_ concentrations (hourly means) at the roadside and distal playground (7 to 15 m from roadside) for the four schools (SI Fig. S2), over the monitored summer/autumn interval (June to October 2019, Cambri sensor network; data summaries in SI Figs. S3–14), together with vehicle count data (2019) for each location. SI Figs. S3 and S4 show the PM_2.5_ and PM_1_ concentrations for the same interval. Monthly roadside PM levels were highest in August/September and lowest in July (SI Table S1).

**Figure 2:**
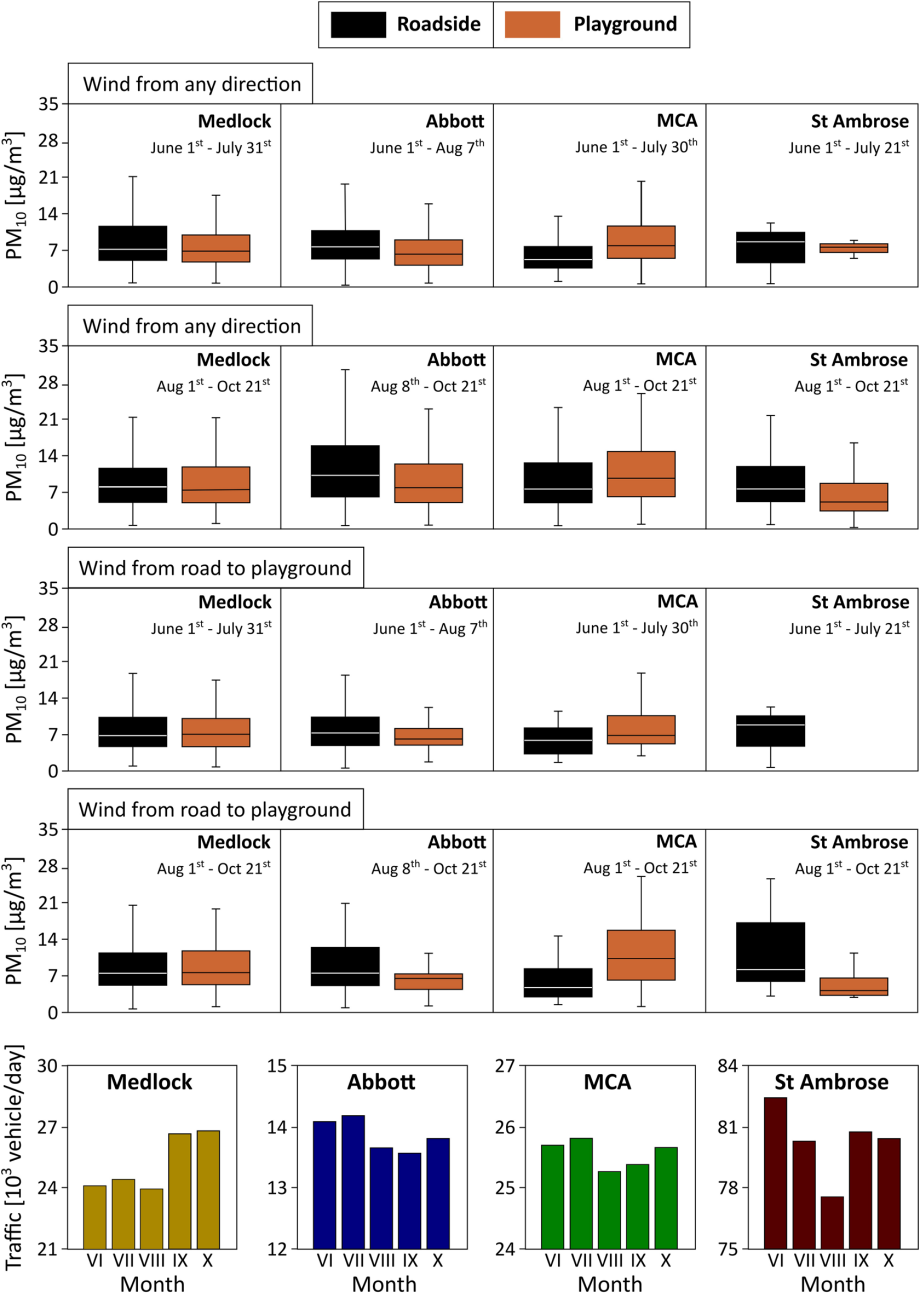
PM_10_ concentrations (hourly average) for summer (pre-tredge installations) and late summer/autumn, 2019 at the roadside and at distal playground (14–17 m from roadside for Medlock, Abbott and St Ambrose, 7 m for MCA), and the road traffic volume for the 4 schools. In the box-whisker plots, boxes indicate median value, lower and upper quartiles, and whiskers show 10th and 90th percentiles. The absence of data (St Ambrose summer) reflects sensor malfunction.

The least heavily-trafficked school location, Abbott, experienced the highest levels (averaged over the 5-month measurement period) of PM_10_ (~10.4 µg/m^3^) and PM_2.5_ (~6.4 µg/m^3^), compared to 8.8 µg/m^3^ (PM_10_) and 6.3 µg/m^3^ (PM_2.5_) for Medlock, 8.3 µg/m^3^ (PM_10_) and 6.1 µg/m^3^ (PM_2.5_) for St Ambrose, and 8.0 µg/m^3^ (PM_10_) and 6.2 µg/m^3^ (PM_2.5_) for MCA. Similar levels of PM_1_ were noted for all the schools, ~3.1 µg/m^3^ at Medlock, Abbott and MCA, and ~2.9 µg/m^3^ at St Ambrose. Co-location of a Cambri sensor at the University of Manchester air quality supersite indicates these measured PM_10_, PM_2.5_, and PM_1_ concentrations are lower, on average, by ~36%, 15% and 69%, respectively, than those recorded by a certificated aerosol spectrometer (Palas FIDAS 200 E). The FIDAS, in turn, was calibrated according to the UK MCERTS specification for particulate matter, and audited by the UK National Physical Laboratory.

The measured PM_2.5_ concentrations exceeded the WHO’s (2021) recommended annual mean limit (5 µg/m^3^) for all schools. For PM_10_, the measured average levels were within the current recommended limit (annual mean 15 µg/m^3^) for all schools. Notable, however, is the very high temporal variability of PM at each school, reaching high but short-lived peaks (up to even 16 × higher than the average values). For example, the highest PM_10_ value (hourly average) was observed at MCA on July 24th 2019 (74.2 µg/m^3^), and highest PM_2.5_ (64.2 µg/m^3^) at MCA on September 22nd 2019, and PM_1_ (50.2 µg/m^3^) at MCA on July 24th 2019.

### PM concentrations—roadside and playground

Tredges were installed at three of the schools during the school holidays (July/August 2019). An ivy screen was installed at Abbott School; western red cedar at St Ambrose School; a roadside tredge of alternating western red cedar/Swedish birch and an inner juniper hedge at MCA. Medlock, the ‘control’ school (i.e., no tredge installation), demonstrates negligible distance decay effect in roadside vs playground PM concentrations throughout the monitored interval (Fig. 2). Abbott and St Ambrose schools similarly display little roadside/playground differences in PM concentrations in the summer period, prior to tredge installation. (The ‘playground’ sensor at MCA was affected by an air conditioning exhaust outlet, and recorded consistently increased PM values compared with roadside; hence, omitted from further consideration here). After tredge installation, Abbott (ivy screen) displays a small reduction in playground PM concentrations, while St Ambrose (western red cedar) displays a larger PM reduction (Fig. 2; SI Figs. S3 and S4). These playground PM reductions were greater when the wind direction was from the road to the playground (Fig. 2; SI Figs. S3 and S4). The significance of these measured roadside/playground PM reductions is uncertain, however, given the estimated between-sensor uncertainties (which range from 22 to 31% for PM_10_, 10–15% for PM_2.5_, and 9–14% for PM_1_, SI Table S2).

**Figure 3:**
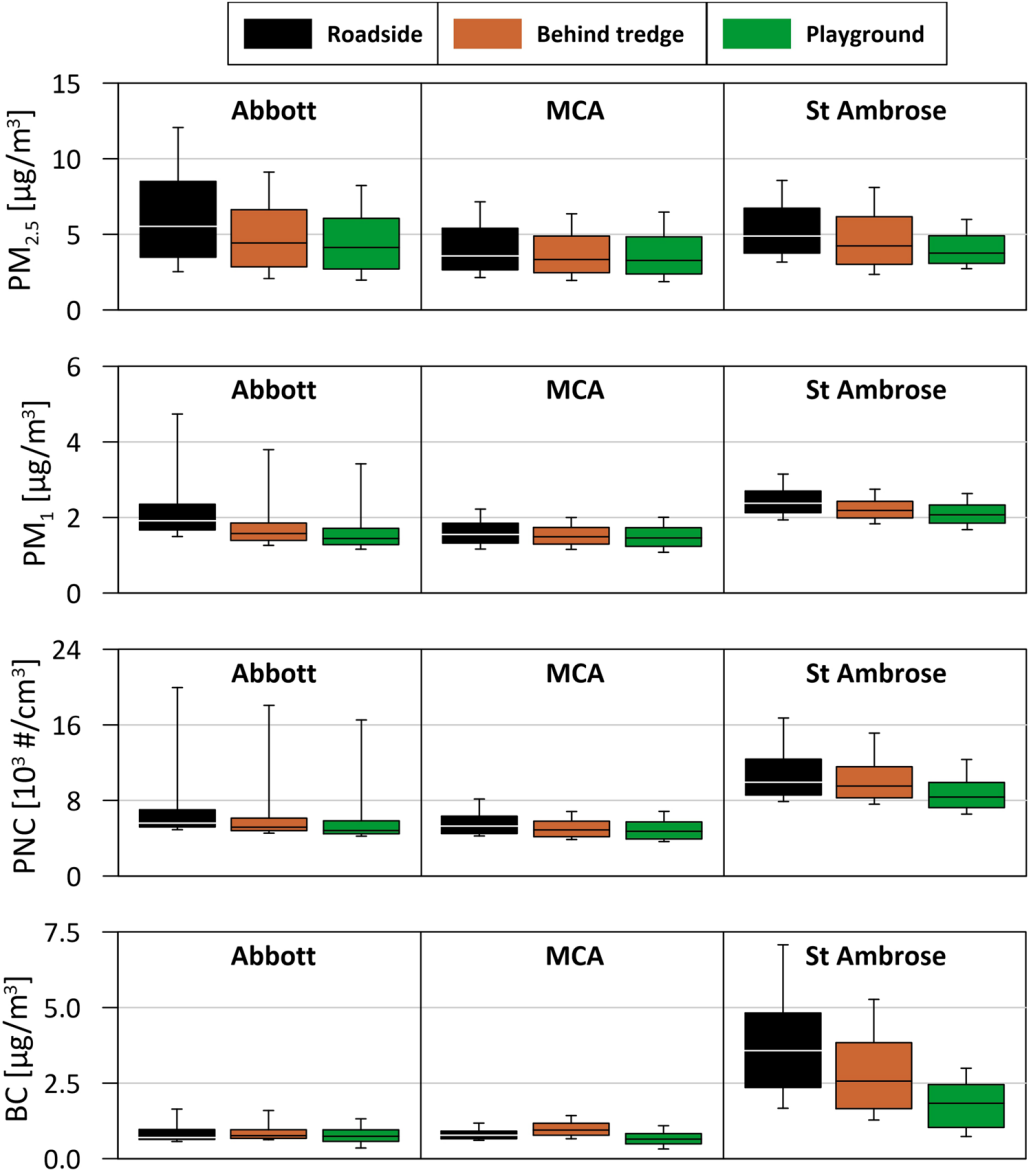
Black carbon (BC), PM_2.5_, PM_1_ and particle number concentrations (PNCs), 1-min aves., measured at the roadsides, and in the playgrounds at 1 m (‘Behind tredge’) and ~ 5 m (‘Playground’) behind the tredges. Boxes indicate median value, lower and upper quartiles, and whiskers show 10th and 90th percentiles.

Hence, additional air quality measurements were made (Fig. 3). Black carbon (BC) was measured simultaneously at roadside and playground, at the three schools with tredges, in November 2020, and PM_2.5_, PM_1_, and particle number concentrations (PNCs) measured (portable optical particle spectrometers, POPS) simultaneously at the roadsides, and in the playgrounds at 1m and ~ 5m behind the tredges in April 2021, after further growth of the tredges. Between-sensor uncertainty estimates for the BC measurements range from 2 to 12%, and for the POPS measurements from 1 to 6% (SI Table S3).

BC values are significantly (~ 5×) higher for the most heavily-trafficked site, St Ambrose (Fig. 3) (t-test; p < 0.05). The high and variable PM concentrations but lower BC (and NO, SI Fig. S11) values measured at Abbott may reflect emissions from non-road traffic sources; a 6-track railway line lies ~ 100 m to the west (SI Fig. S2). Bivariate polar plots indicate high PM concentrations at Abbott are associated with moderate westerly winds (SI Fig. S14).

PM_2.5_ and PM_1_ concentrations, measured by the POPS instruments, are in good agreement with those from the Cambri sensors (Figs. 2, 3; SI Figs. S3–10). For the roadside sensors, PNCs were highest for St Ambrose (median ~9877 #/cm^3^), followed by Abbott (~5545 #/cm^3^), and MCA (5234 #/cm^3^) (Fig. 3; SI Table S3). At all three schools, measurable (and statistically significant; p < 0.05) reductions in PM_2.5_, PM_1_, and PNC were observed in the playgrounds (Fig. 3; Table S3). The measurements indicate greatest playground PM reductions by the ivy screen; ~25% for median PM_2.5_ and PM_1_, 14% for PNC, but 5% increase in black carbon (BC). Behind the evergreen western red cedar tredge at St Ambrose, the playground reductions were 23% for median PM_2.5_, 13% for PM_1_, 16% for PNC, and 49% for BC. Moreover, the tredges substantially reduced the magnitude and frequency of acute ‘spikes’ in PM and BC concentrations; for example, by 67–82% for PM_2.5_, PM_1_ and PNC in the St Ambrose playground (SI Table S3). At MCA, behind the outer tredge (alternating western red cedar/Swedish birch) and inner row of juniper, the playground reductions are lower; 9%, 6%, 11% and 16% for PM_2.5,_ PM_1_, PNC and BC, respectively (SI Table S3).

### Leaf magnetic loadings and PM deposition estimates

**Figure 4:**
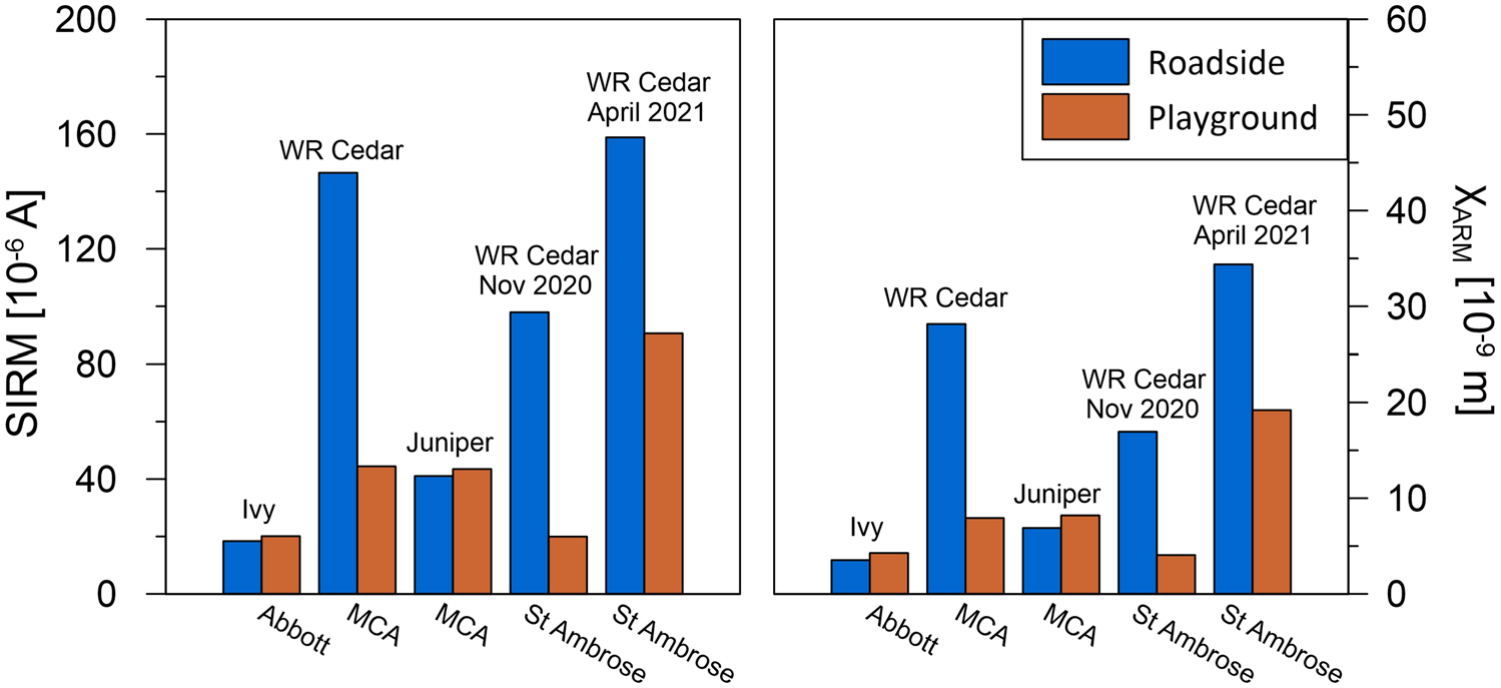
Surface area-specific saturation remanent magnetisation (SIRM) and susceptibility of anhysteretic remanent magnetisation (χ_ARM_) of leaf samples taken from the roadside and playground sides of the tredges.

‘Clean’ leaves should display negligible magnetic remanence, because of their high content of carbon and water (which display diamagnetic behaviour, i.e., negative magnetic susceptibility, zero magnetic remanence). However, traffic-derived PM always contains strongly magnetic particles, produced especially by brakewear^31,67^, vehicle exhausts, tyrewear etc. Deposition of magnetic PM on leaves makes them variably magnetic^68^. The amount of magnetic PM deposited on our tredges was quantified by measuring the saturation remanent magnetisation (SIRM) of leaf samples from the roadside and playground sides of the tredges (Fig. 4). Western red cedar leaves (St Ambrose, and MCA) collected the greatest amounts of magnetic grains, as measured by SIRM ranging between 98 × 10^−6^ A and 159 × 10^−6^ A (roadside). For these cedar tredges, the roadside SIRM values were 2–5× higher than the playground side (Fig. 4). Much lower SIRM values were obtained for the ivy leaves at Abbott (18 × 10^−6^ A roadside) and the inner juniper tredge at MCA (41 × 10^−6^ A roadside), and no decrease in leaf SIRM was observed from the roadside to the playground side for these species (Fig. 4).

We also measured another type of magnetic remanence, anhysteretic remanent magnetisation (ARM), which is sensitive to magnetic particles ~25 nm in size. As with SIRM, highest χ_ARM_ values were obtained for western red cedar at St Ambrose (17–34 × 10^−9^ m) and MCA (28 × 10^−9^ m), with significant decrease (× 2–4) for the playground sides (Fig. 4). The ivy at Abbott and juniper at MCA collected much lower amounts of ultrafine magnetic grains (χ_ARM_ from 4 to 7 × 10^−9^ m), with no reductions from the roadside to the playground sides. PM deposition on ivy leaves has been reported^69,70^ to be lower (e.g., 8–14 µg/cm^2^; up to 30 µg/cm^2^^61^) than numerous other broadleaved and coniferous species, which display leaf PM loadings of > 50–230 µg/cm^2^ (e.g.^61,71^). While the SIRM and χ_ARM_ values varied for the leaves at the three schools, the SIRM/χ_ARM_ ratio was relatively constant, indicating a similar ferrimagnetic grain size population at each site (SI Table S4). Electron microscopy images (e.g., SI Fig. S1) show that the majority of leaf-deposited pollution particles are < 1 µm in size (e.g.^34,71^).

The leaf magnetic loadings can be used to estimate the total amount of PM_10_ deposited on the leaves and its proportion compared with the total traffic-emitted PM_10_ (SI Table S5). Given, for example, the measured SIRM at St Ambrose (98–20 × 10^−6^ A for leaves collected in November 2020), SIRM values for synthetic magnetite of 5.0 Am^2^/kg (for magnetite grains 0.1–1 µm ^72^), average mass concentrations of magnetite in roadside dust (0.32–0.95 wt.%; data for a highly-trafficked street in Birmingham, UK^31^), and total leaf surface area (2.5 m^2^/tree), the estimated leaf PM mass is ~ 124 µg/cm^2^ and the total mass of PM_10_ deposited on a tree is 14.7–22.1 g/tree. These leaf PM mass estimates are comparable with those measured^71^ for four evergreen species, which ranged from ~72 µg/cm^2^ (in *P. tabulaeformis*) to 232 µg/cm^2^ (in *J. formosana*). The tredge at St Ambrose comprises 102 western red cedar trees so an estimated 158–705 g were deposited on the whole tredge.

For a road section 100 m in length, average traffic count of 80,000 vehicles/day (Fig. 2), and estimated PM_10_ emission factor of ~100 mg/(km vehicle)^56^, the average emission mass flow is ~9.3 mg/s. PM deposition on leaves may reach ‘saturation’/equilibrium over ~ 6 dry days^35^ but PM is washed off leaves during rainfall events. PM wash-off from leaves varies with species, leaf traits and rainfall intensity/duration but removal of 40−80% of surface-deposited PM has typically been reported for a variety of species (e.g.^63,73–75^). In November 2020, the tredge leaves were sampled 3 dry days after a rainfall event. Over that time period, therefore, the estimated cumulative traffic-emitted PM_10_ mass is ~2400 g. Assuming the prior rainfall removed either 50% or 75% of the previously-deposited leaf PM, then comparing the PM_10_ mass deposited on the whole tredge (102 western red cedar trees) and the cumulative traffic-emitted PM_10_ mass, we can estimate that between ~7% and 29% of the traffic-emitted PM_10_ was deposited on the western red cedar tredge at St Ambrose at this time (SI Table S5).

## Discussion

Given known and suspected impacts of PM exposure on human health, especially for particularly vulnerable population groups (e.g., young children), fast and effective mitigation of exposure levels -even by what is conventionally considered ‘small’ amounts -is essential. In the UK, COMEAP^76^ estimate that reducing the annual average concentration of PM_2.5_ by only 1 µg/m^3^ would save ~3.6 million life years, equivalent to an increase in life expectancy of 20 days in people born in 2008.

Here, air quality measurements in open road contexts demonstrate that installation of carefully-selected and designed vegetation structures (‘tredges’) at the school roadside can rapidly achieve significant reductions in playground PM concentrations. For the ivy screen (Abbott), the measured PM_2.5_ and PM_1_ reductions probably dominantly reflect some blocking of the dominantly westerly airflow and subsequently reduced leeward PM, since PM deposition on the ivy leaves (as measured by leaf magnetic remanences) was low. In contrast, PM deposition on the evergreen western red cedar tredge (St Ambrose) was much greater; leaf magnetic loadings indicate that up to 29% of the local traffic-emitted PM_10_ was deposited on the western red cedar tredge at St Ambrose at the time of sampling (Nov. 2020). It is reasonable to assume that most of the leaf-deposited PM originated from the proximal, traffic-related sources (rather than the regionally-dispersed PM). Since the local, traffic-derived PM increment typically constitutes ~ 50% of measured ambient PM, then leaf deposition at St Ambrose removed ~ 46% and 26% of the traffic-sourced PM_2.5_ and PM_1_, respectively. Further, and possibly of major importance for child health, the tredges substantially reduced the magnitude and frequency of acute ‘spikes’ in PM and BC concentrations; for example, by 67-82% for PM_2.5_, PM_1_ and PNC in the St Ambrose playground. The St Ambrose tredge also produced marked reductions in average playground BC concentrations, unlike the other two tredges. Notable is that the playground air quality improvements at St Ambrose were achieved with a narrow (~1 m) tredge (i.e. 10% of the width recommended by e.g.^77^). This is important; in many urban situations, space for planting roadside tredges is often very limited. The effectiveness of the inner juniper hedge and outer birch/western red cedar tredge (MCA) was less but likely to increase with time (these plants had experienced sparse growth at the times of sampling).

These findings demonstrate that roadside vegetation can be designed, installed and maintained to achieve rapid, significant and cost-effective improvement of air quality by optimising PM deposition on plant leaves. Critical to air quality improvement through PM deposition is selection of species with leaf traits creating high *Vd* values; combined with high rates of PM removal by rainfall and/or watering wash-off (to restore leaf PM-capturing ability), and permeability (to avoid blocking of PM transport and increased roadside PM). Fowler et al.’s^64^ *Vd* value of 1.07 cm s^−1^ for urban ‘woodland’ (mixed oak, sweet chestnut, beech, holly) was described recently as ‘high’^78^. Yet, as independently attested by their much higher leaf PM loadings (e.g.,^61,71^), numerous other species demonstrate higher *Vd* values (e.g.,^35^). Hence, substantively higher *Vd* values need to be included in any modelling study seeking to quantify roadside vegetation and leaf PM deposition effects (e.g.,^44,57^). The air quality improvements measured here were obtained in open road contexts but it is equally important to incorporate realistic/optimised *Vd* values in street canyon modelling, to inform any removal of existing (too high/large) vegetation and/or installation of new, optimised vegetation.

To trap PM before its dispersion into the regional airshed, tredges (with near-ground level leaves) should be close to the local, traffic-emitted sources. Tredge height should be managed, around adult head-height and certainly never exceeding the height of any adjacent buildings, to prevent reduced airflow and upward dispersion which large, tall trees can induce, thus worsening roadside pollution^44^. Under dry conditions, watering of tredges might be required, to wash off leaf-accumulated PM into the soil below and renew their leaf PM-capturing capability.Other benefits accrue from roadside greening (e.g., green space wellbeing, noise reduction, visual appeal, safety, privacy, biodiversity benefits, climate change proofing) but species selection must also preclude deleterious effects (e.g., excessive production of allergenic pollen, poisonous fruit/seeds, volatile organic compounds).

Here, the measured amounts of leaf-deposited magnetic PM after tredge installation, and the measured reductions in playground PM, PNC and BC, demonstrate that air quality improvements by deposition can be achieved at the local, near-road, community/playground scale. Modelling/quantification of the complexity of factors (dispersion- and deposition-related) influencing these playground PM concentrations, whilst desirable, would have required substantial additional resources. Beyond this study, and given the importance to human health of immediate exposure reduction, integrated experimental and modelling investigations of roadside greening effects are timely and important. Ideally, robust measurement of species-specific *Vd* and permeability values under different meteorological and pollution conditions would be combined with iterative modelling of airflow/dispersion and size-specific PM deposition effects. Essential for such investigations are sufficient resource to implement simultaneous, multi-site, robust measurements of PM and PNC (and detailed characterisation of airborne and leaf-deposited PM), combined with sufficient computational resource for model runs incorporating multiple iterations with varying *Vd *^57^, rather than/as well as leaf area index and density^79,80^, permeability and meteorological (especially wind speed) parameterisations. Essential is that vegetation effects are modelled at the micro- as well as the macro-scale^44^. 

Finally, if sufficient, well-designed roadside tredges were installed and managed appropriately along heavily-trafficked streets across an urban area, leading to reduction in the amount of PM leaving those road areas, then PM loadings at the city and regional scale should also fall (albeit with the effect of concentrating pollutants in the soil beneath the tredges). As an interim, fast and cost-effective measure (i.e., prior to policy-driven reductions of PM emissions), and for protection of brain, heart and pre-natal health, careful use of roadside tredges should be considered for widespread implementation. City-wide modelling of the air quality impacts of such structures would be of timely value in this context. 

## Methods

In this study in Manchester, U.K., we investigated the possibility of using optimised green infrastructure -instantly installed ‘tredges’ (^58^) -to achieve measurable improvements in air quality (PM concentrations) in the playgrounds of four primary schools located adjacent to major arterial routes through the city. These schools are sited within the Greater Manchester Air Quality Management Area (AQMA), where air pollution standards are unlikely to be met.

For air quality monitoring over a period of several months, Cambri sensors (Shenzhen Cambri Environmental Technology Ltd., China) were installed at the roadside and > 10 m from the roadside. Particles with diameters between 0.37 and 17.4 μm were detected using the Alphasense OPC-N2 sensor. The sensors were co-located for cross-calibration of PM_10_, PM_2.5_, and PM_1_ measurements (via orthogonal regression of data and sensor 1 h or daily means), and estimation of between-sensor uncertainties, before (May 2019) and after (October/November 2019) the monitored interval. A Cambri sensor was also co-located at Manchester University’s Air Quality Super Site. The PM data are presented as 1-h average values. For safety reasons, the playground sensors were required to be sited distally (14–17 m) from the road at the rear of the playground areas (for Medlock, St Ambrose and Abbott schools) and close to the school frontage (7 m from the road, MCA school). These locations necessitated use of car batteries to power the sensors; battery failures led to sensor drift and episodic malfunction (creating gaps in data coverage). The PM values recorded by the distal MCA sensor were consistently affected by a nearby air conditioning exhaust outlet.

Black carbon (BC) was measured (MicroAeth M350) simultaneously at roadside and playground, at the 3 schools with tredges, at morning or evening rush hours in November 2020. Rush hour PM_2.5_, PM_1_, and PNCs were measured (portable optical particle spectrometers, POPS, Handix Scientific; particle diameter measurement range between ~ 0.13 to 3.0 µm) simultaneously at roadside and playground at 1 m and ~ 5 m behind the tredges in April 2021, after further growth of the tredges. The POPS and aethalometers were cross-calibrated (co-location at end of the measurement periods), by orthogonal regression of the sensor data vs the sensor mean values (1 min ave.), and uncertainties estimated (https://ec.europa.eu/environment/air/quality/legislation/pdf/equivalence.pdf).

Between-sensor uncertainty estimates for the BC measurements range from 2 to 12%, and for the POPS measurements from 1 to 6% (Table S3).

For quantification of magnetic PM loadings, leaves were sampled from the road and playground side of the tredges and measurements made (at the Centre for Environmental Magnetism & Palaeomagnetism, Lancaster University) of their anhysteretic remanent magnetization (ARM) and saturation remanence (SIRM). ARM is sensitive to the presence of ferrimagnetic particles with a mean particle size of ~ 25 nm. The SIRM indicates the total concentration of magnetic particles on the leaves. ARM was induced using a Molspin A. F. demagnetiser, with ARM attachment, generating a dc biasing field (0.08 mT) in the presence of an alternating field (100 milliTesla (mT) peak field). The ARM was measured using a spinner magnetometer (JR-6A, AGICO). The susceptibility of ARM (χ_ARM_) was calculated by normalizing the ARM by the dc biasing field. Room temperature remanent magnetization (SIRM) was acquired in a dc field of 1 T, using a Molspin pulse magnetizer. Calibration of the magnetometer was performed using a cross-calibrated rock sample (56.05 × 10^−8^ Am^2^). The average value of each magnetic parameter was normalised for the leaf surface area (in m^2^). The total leaf surface area was measured for two western red cedar trees, using a Li-Cor Model 3100 area meter, with extrapolation to the installed trees via comparison of their height and trunk diameter at chest height.

## Supplementary Information

Supplementary Information.

## Data Availability

The published data are available from the corresponding author.
