# Supplementary material for: Protecting playgrounds: local-scale reduction of airborne particulate matter concentrations through particulate deposition on roadside ‘tredges’ (green infrastructure)
Source: Sci Rep. 2022 Aug 20;12:14236. doi: 10.1038/s41598-022-18509-w (PMC9392798; doi:10.1038/s41598-022-18509-w)
Supplement: Supplementary file 1 — Supplementary Information. [file 41598_2022_18509_MOESM1_ESM.docx]

**Supplementary Information**

**Protecting playgrounds: Local-scale reduction of airborne particulate matter concentrations through particulate deposition on roadside ‘tredges’ (green infrastructure).**

Barbara A. Maher1,*, Tomasz Gonet1,^+^ ,Vassil V. Karloukovski^1^, Wang Huixia^2^ and Thomas J. Bannan^3^

1 Centre for Environmental Magnetism and Palaeomagnetism, Lancaster Environment Centre, Lancaster University, LA1 4YQ, U.K.

^2^ School of Environmental and Municipal Engineering, Xi’an University of Architecture & Technology, Xi’an, 710055, Shaanxi Province PRC

^3^ School of Earth, Environmental and Atmospheric Science, University of Manchester, Manchester, UK

^+^Now at Jaguar Land Rover, Gaydon, Lighthorne Heath, Warwick, CV35 0BJ, U.K.

*[b.maher@lancaster.ac.uk](mailto:b.maher@lancaster.ac.uk)


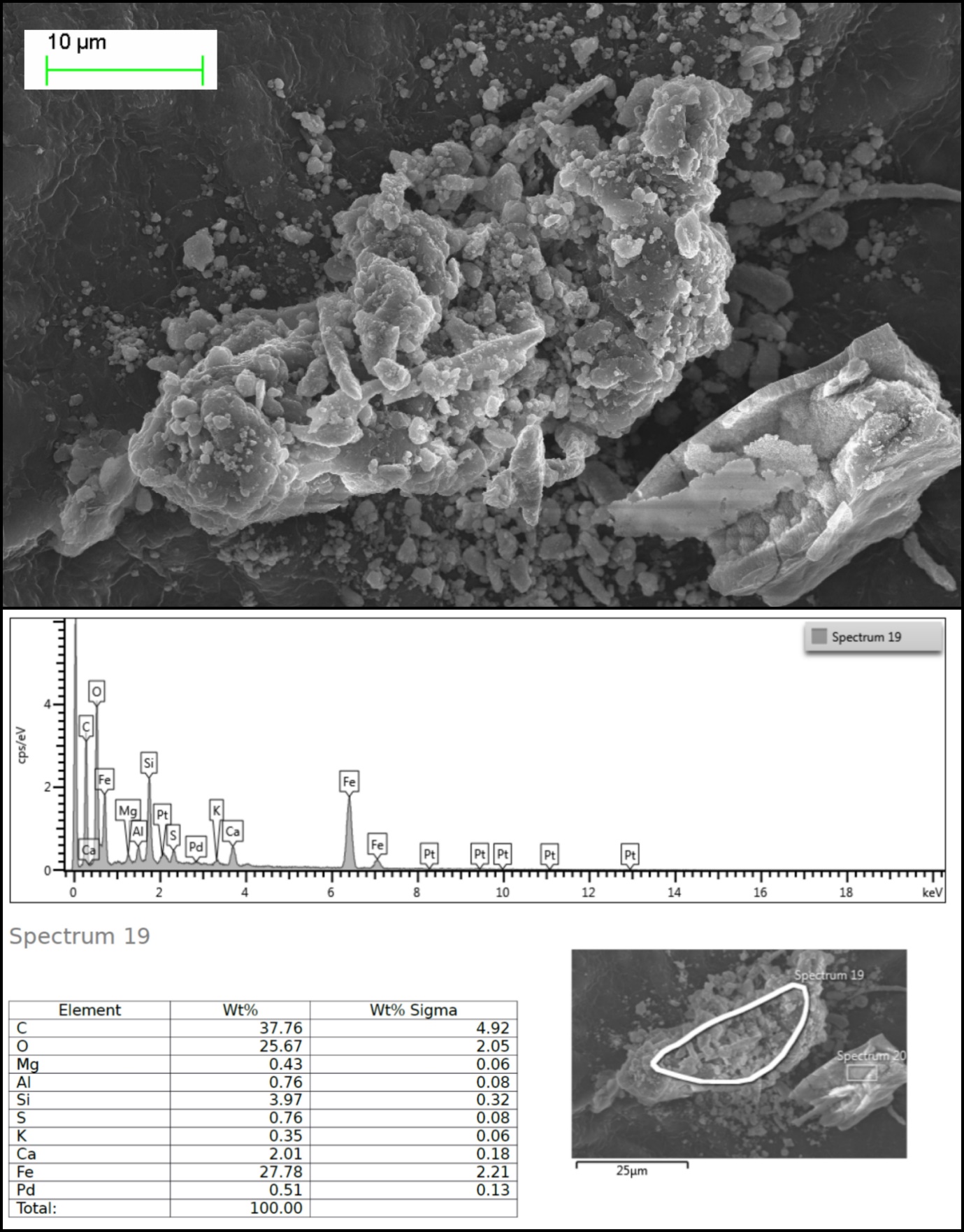


**Figure S1.** Electron microscopy image (5000 × magnification) and energy dispersive spectrum of large agglomerations of fine (iron-rich) particulate matter deposited within micro-furrows on the surface of silver birch leaves (from study by Maher et al., 2013).


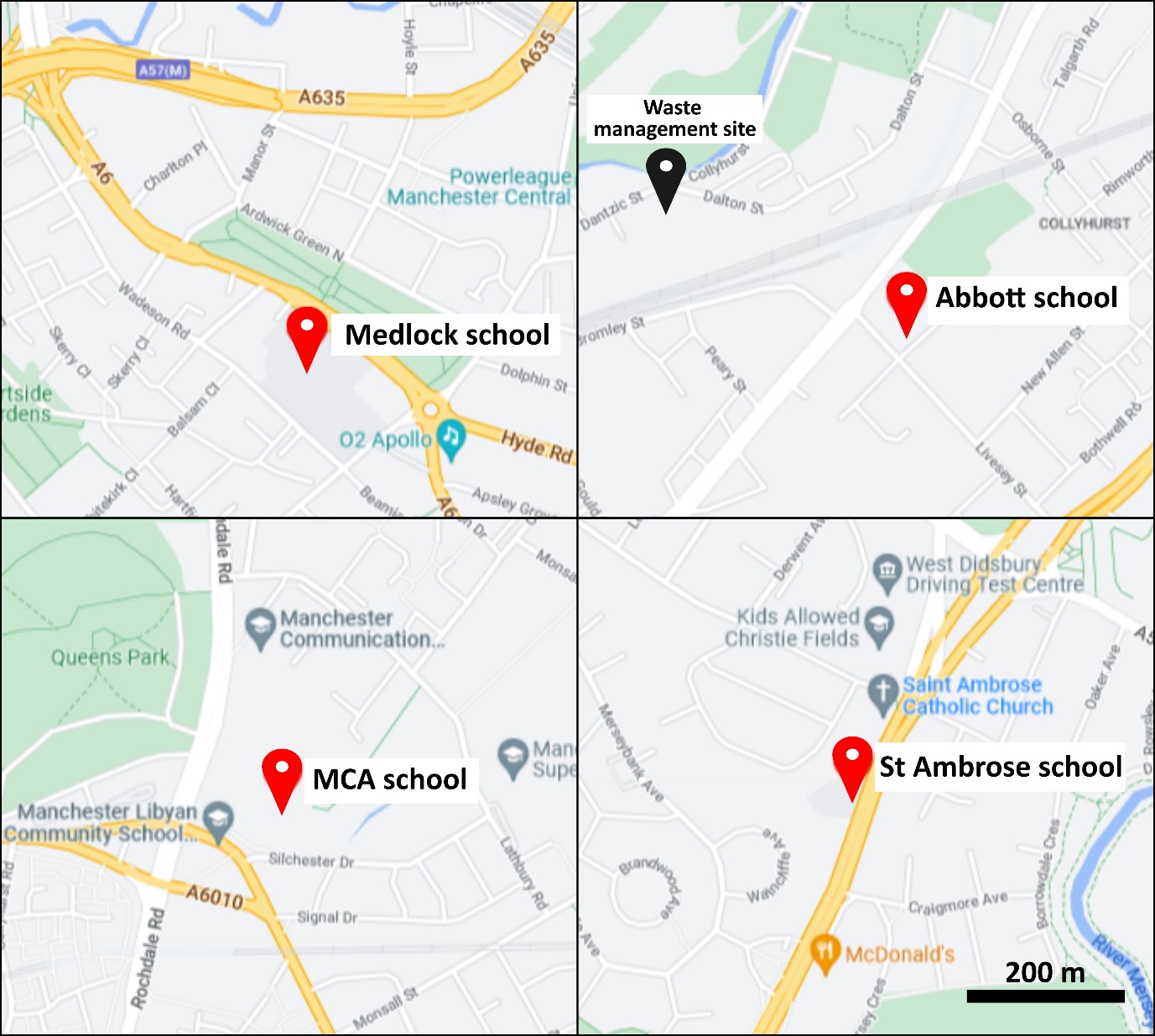


**Figure S2.** Location of the 4 studied schools in Manchester, UK. PM/BC sensors were installed at the roadside and at distal playground (14 −17 m from roadside for Medlock, Abbott and St Ambrose, 7 m for MCA), and the traffic volume for the 4 schools. The tredges were located ~2 m from the roadside at Medlock, MCA and St Ambrose, and ~5 m from the roadside at Abbott.


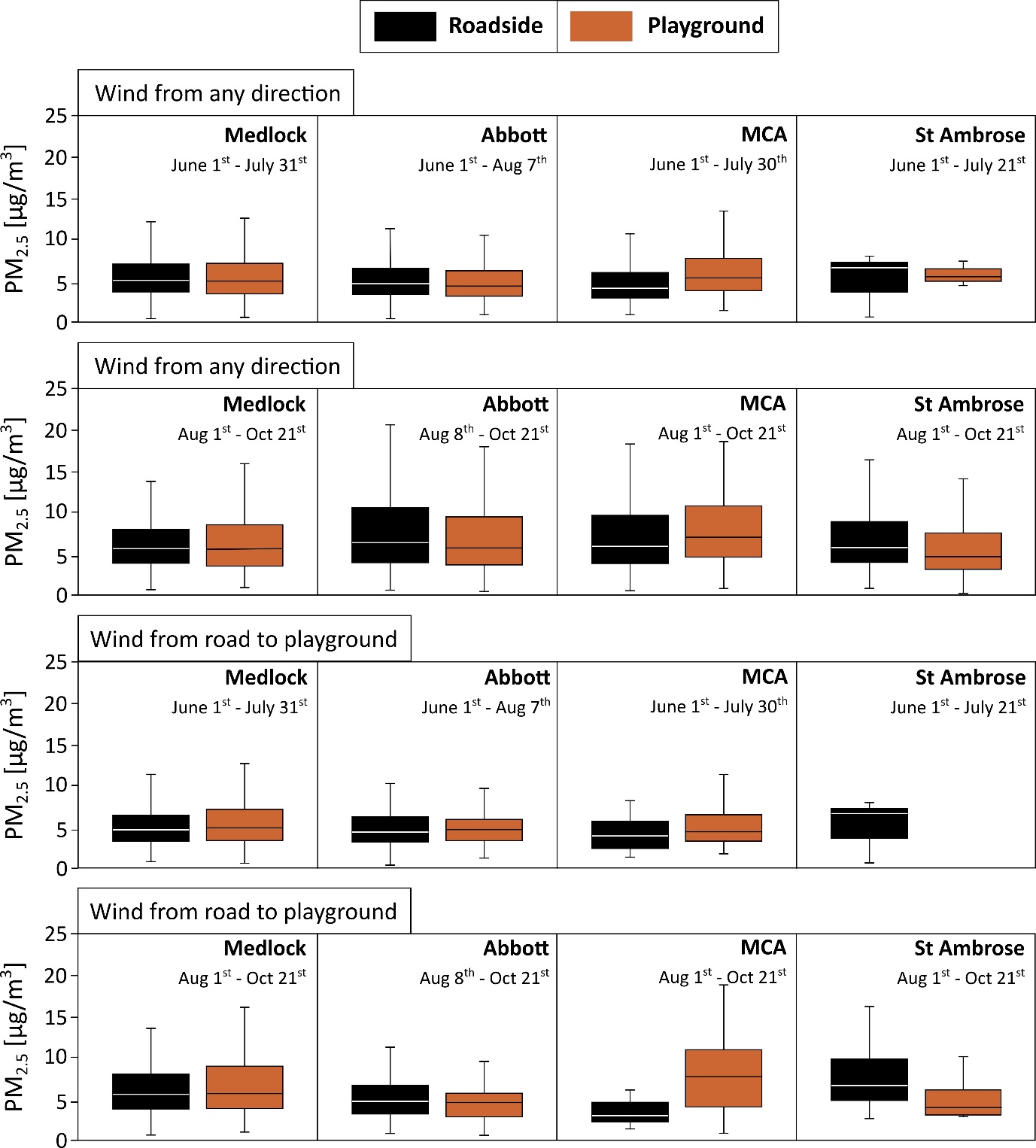


**Figure S3.** PM_2.5_ concentrations (60 min average) for summer (pre-tredge installations) and late summer/autumn, 2019 at the roadside and at distal playground (14 −17 m from roadside for Medlock, Abbott and St Ambrose, 7 m for MCA), and the traffic volume for the 4 schools. In the box-whisker plots, boxes indicate median value, lower and upper quartiles, and whiskers show 10^th^ and 90^th^ percentiles. The absence of data (St Ambrose summer) reflects sensor malfunction. The tredges were installed ~2 m from the roadside at Medlock, MCA and St Ambrose, and ~5 m from the roadside at Abbott.


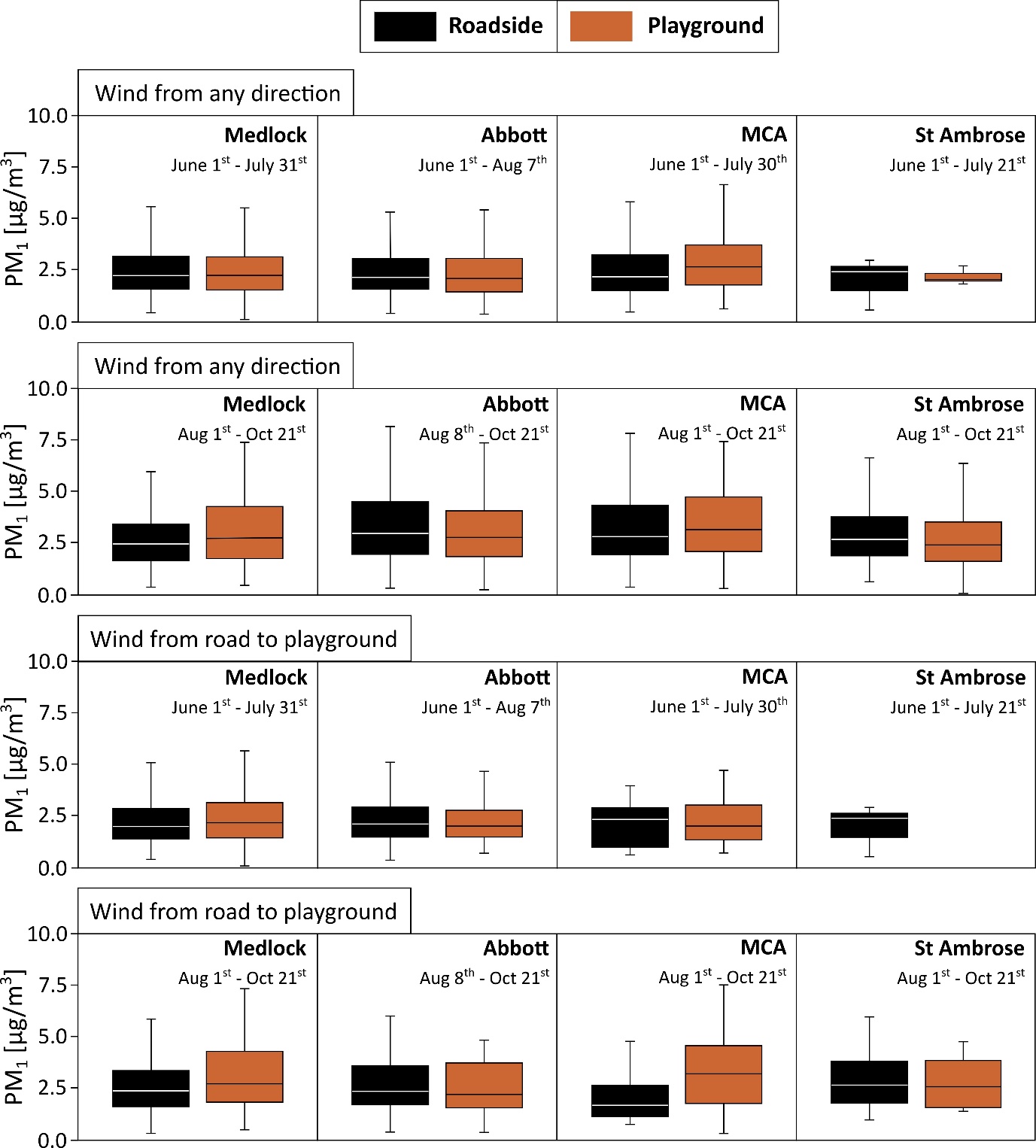


**Figure S4.** PM_1_ concentrations (60 min average) for summer (pre-tredge installations) and late summer/autumn, 2019 at the roadside and at distal playground (14 −17 m from roadside for Medlock, Abbott and St Ambrose, 7 m for MCA), and the traffic volume for the 4 schools. In the box-whisker plots, boxes indicate median value, lower and upper quartiles, and whiskers show 10^th^ and 90^th^ percentiles. The absence of data (St Ambrose summer) reflects sensor malfunction.


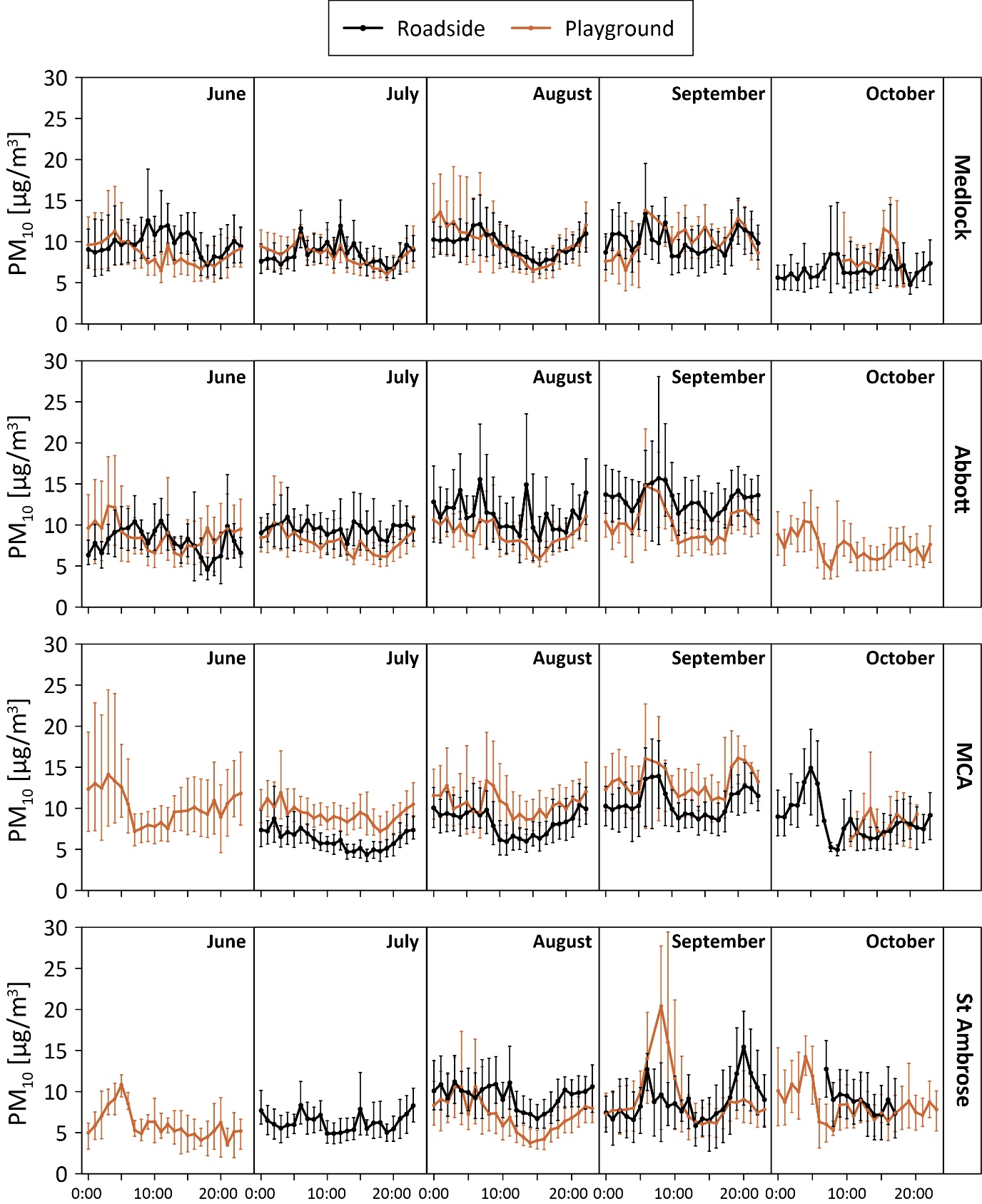


**Figure S5.** Diurnal trends of PM_10_ measured at the roadside and at distal playground (14 −17 m from roadside for Medlock, Abbott and St Ambrose, 7 m for MCA). The absence of data reflects sensor(s) malfunction.


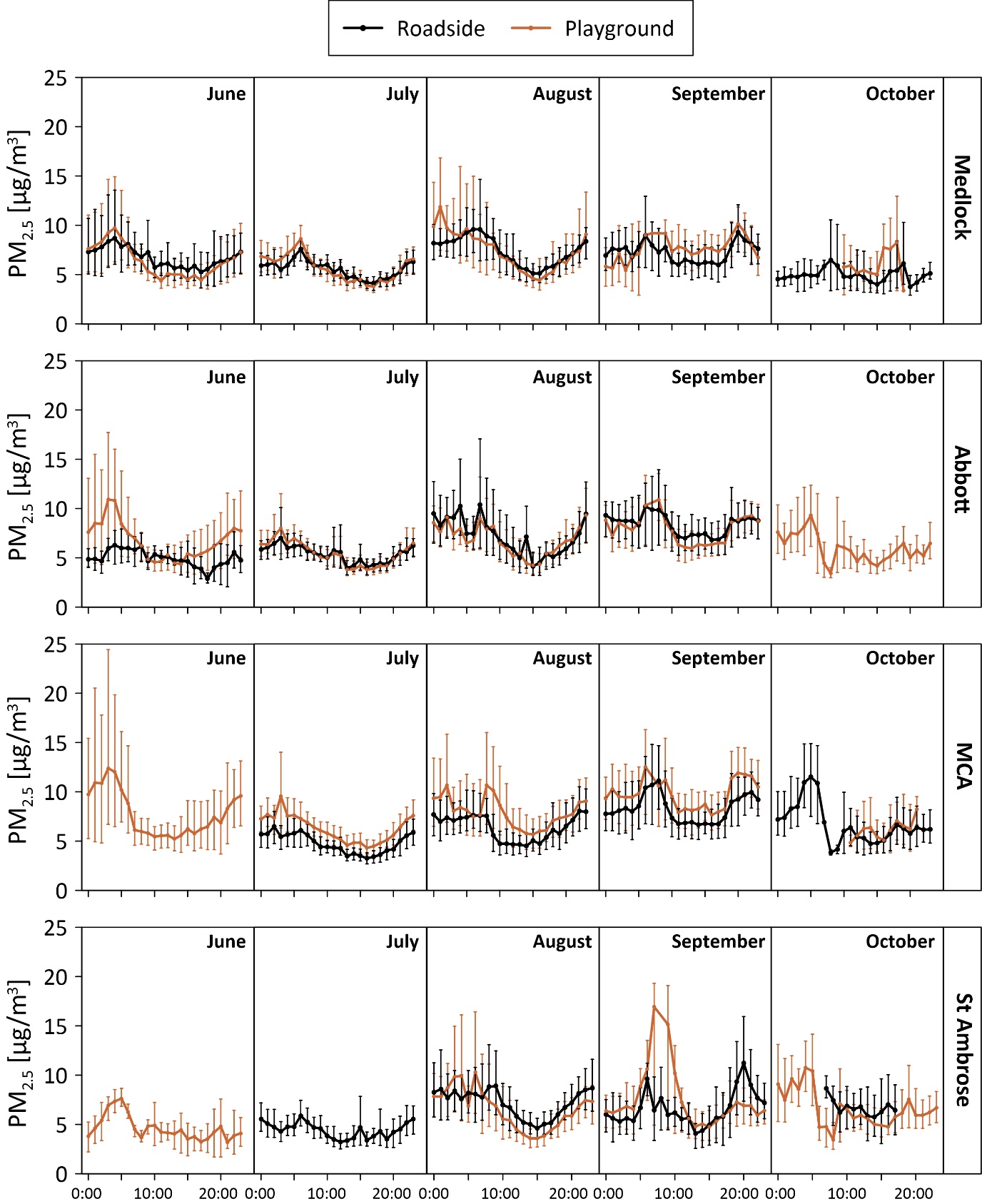


**Figure S6.** Diurnal trends of PM_2.5_ measured at the roadside and at distal playground (14 −17 m from roadside for Medlock, Abbott and St Ambrose, 7 m for MCA). The absence of data reflects sensor(s) malfunction.


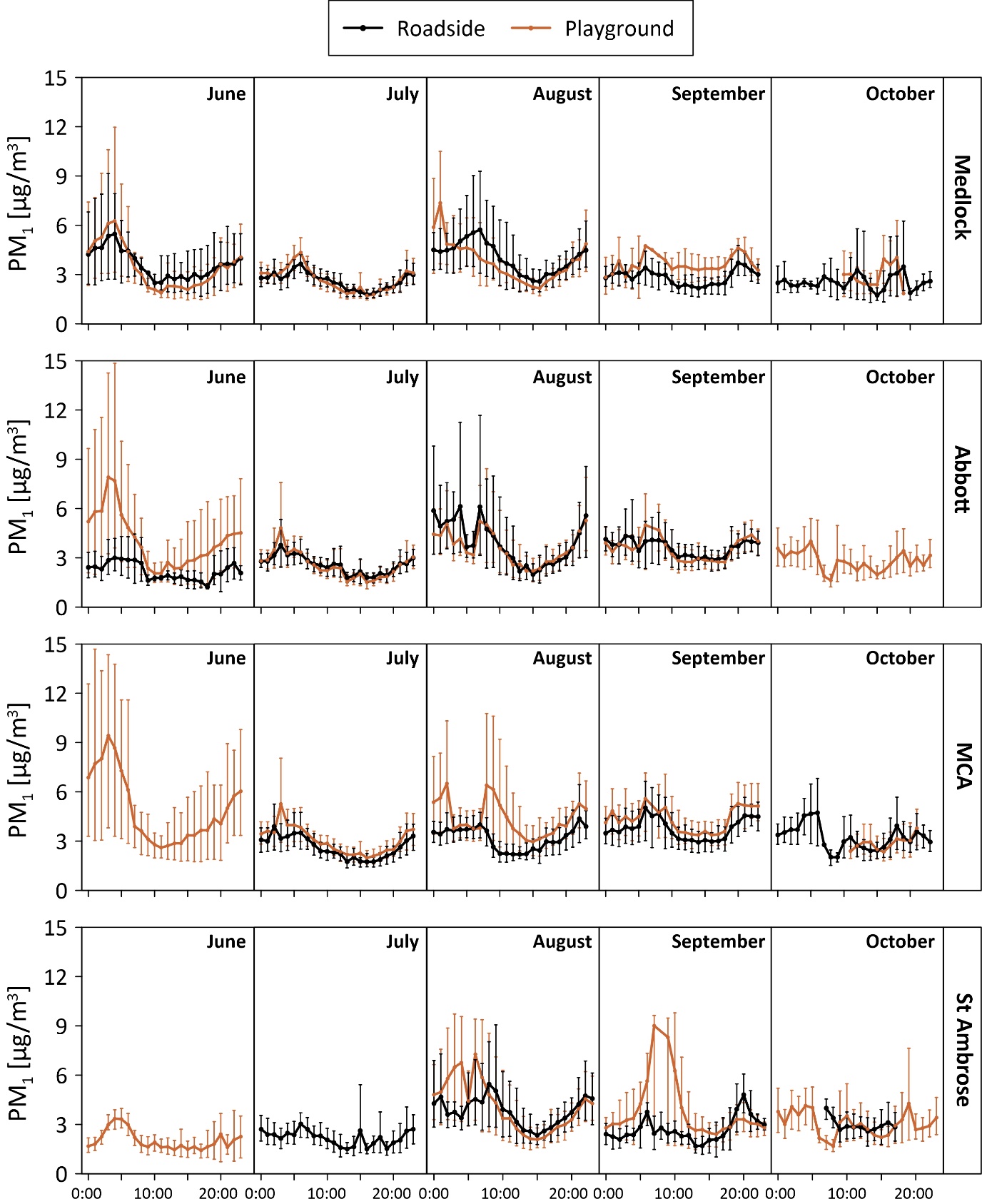


**Figure S7.** Diurnal trends of PM_1_ measured at the roadside and at distal playground (14 −17 m from roadside for Medlock, Abbott and St Ambrose, 7 m for MCA). The absence of data reflects sensor(s) malfunction.


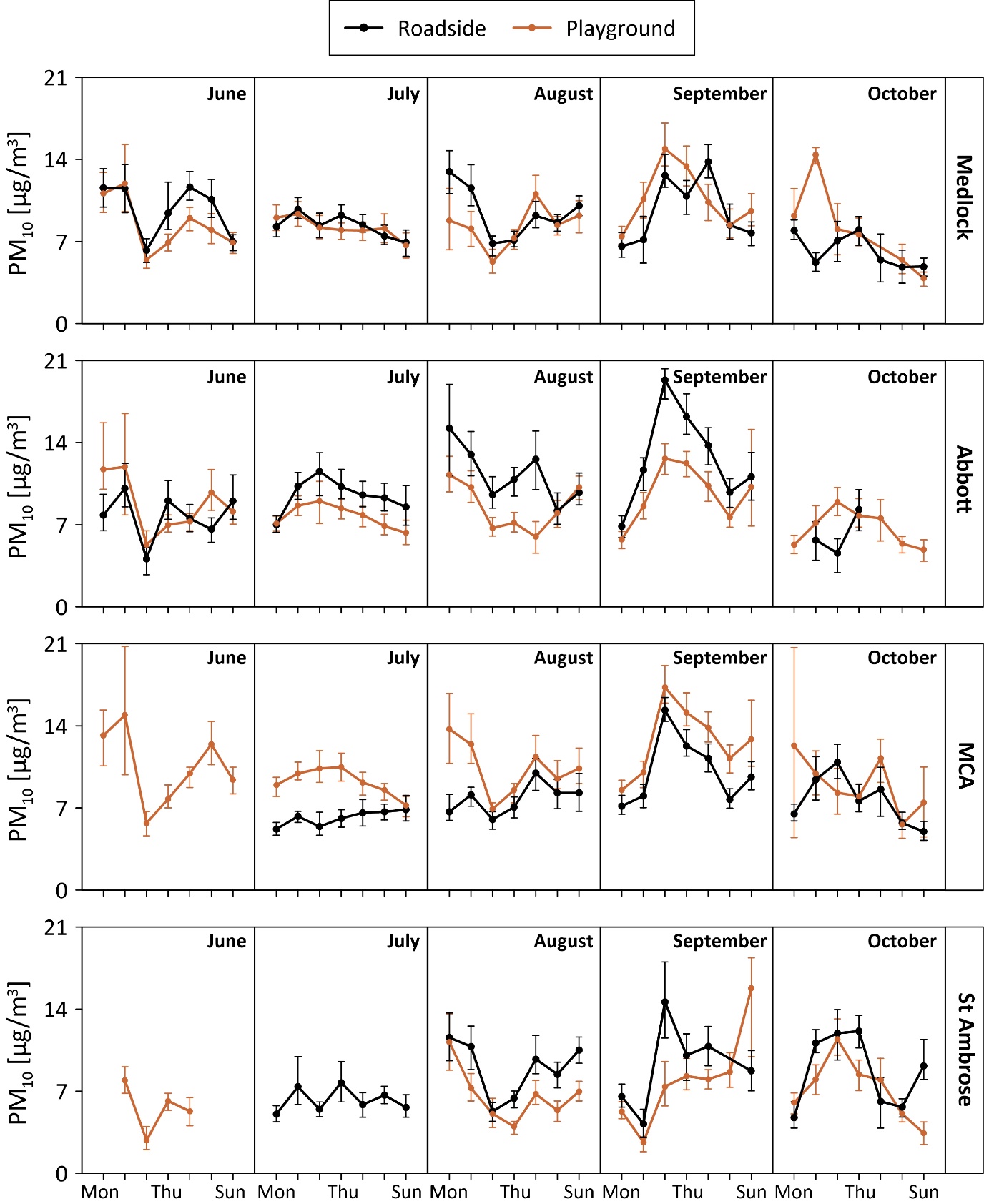


**Figure S8**. Weekly trends of PM_10_ measured at the roadside and at distal playground (14 −17 m from roadside for Medlock, Abbott and St Ambrose, 7 m for MCA). The absence of data reflects sensor(s) malfunction.


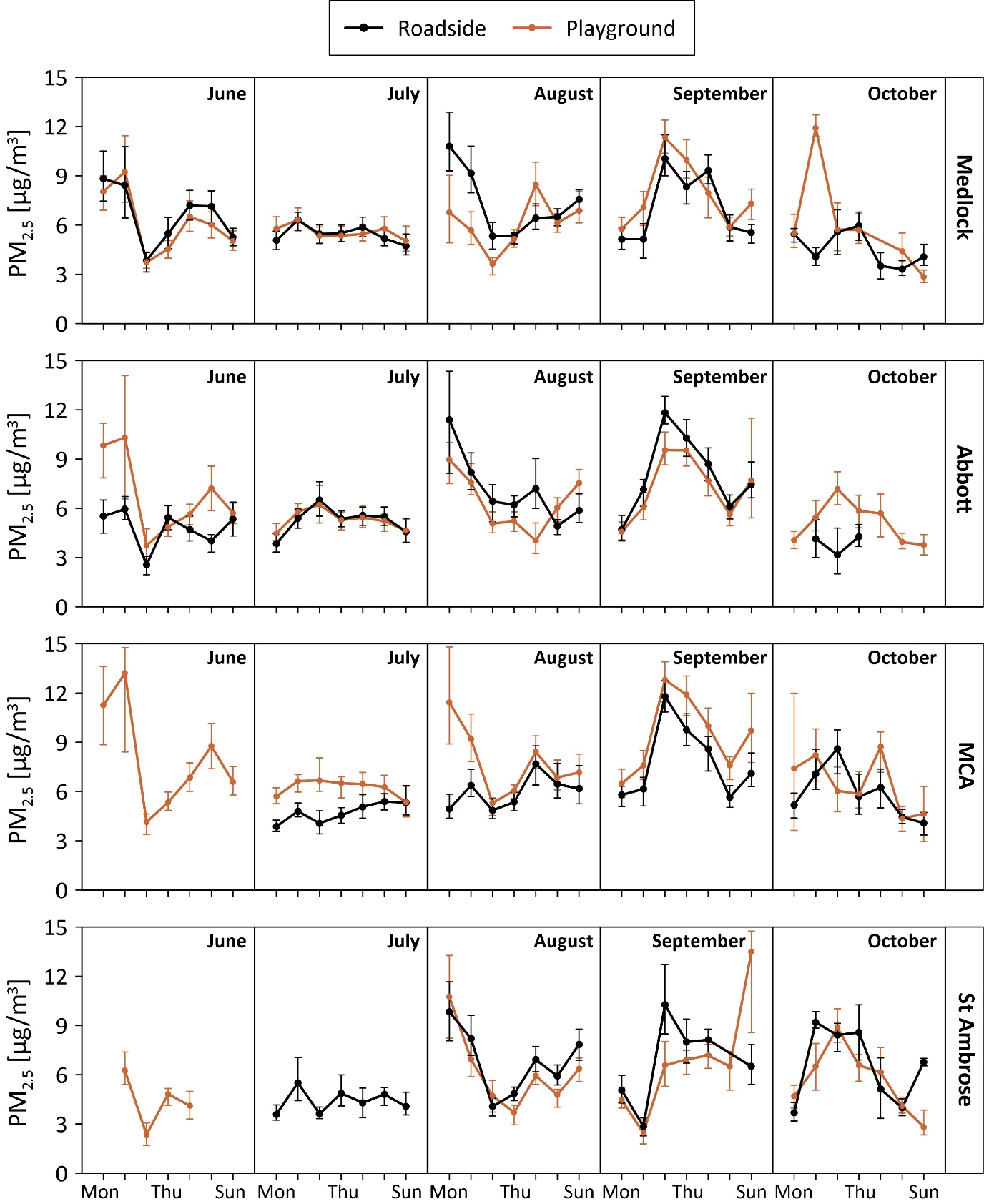


**Figure S9.** Weekly trends of PM_2.5_ measured at the roadside and at distal playground (14 −17 m from roadside for Medlock, Abbott and St Ambrose, 7 m for MCA). The absence of data reflects sensor(s) malfunction.


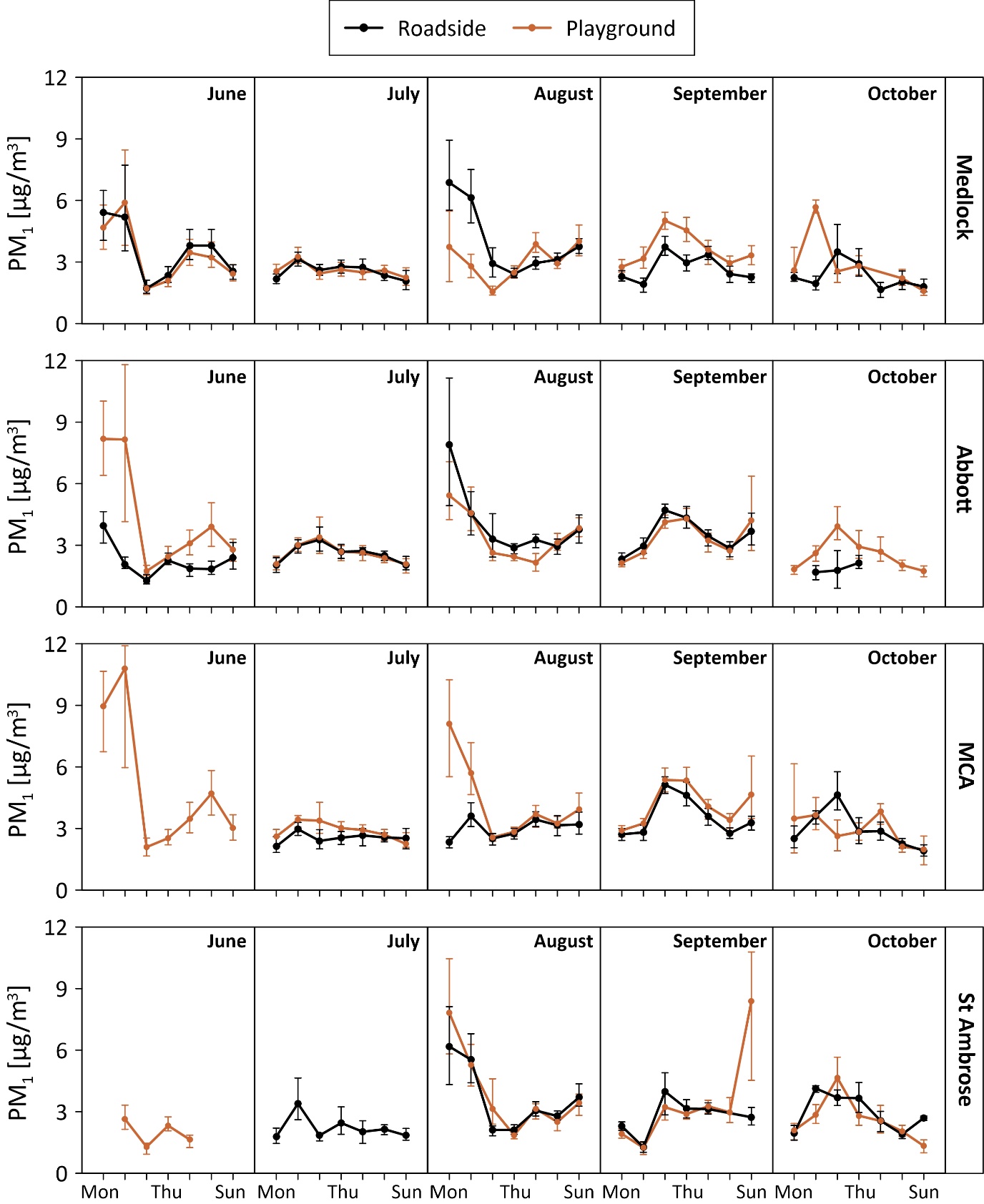


**Figure S10.** Weekly trends of PM_1_ measured at the roadside and at distal playground (14 −17 m from roadside for Medlock, Abbott and St Ambrose, 7 m for MCA). The absence of data reflects sensor(s) malfunction.


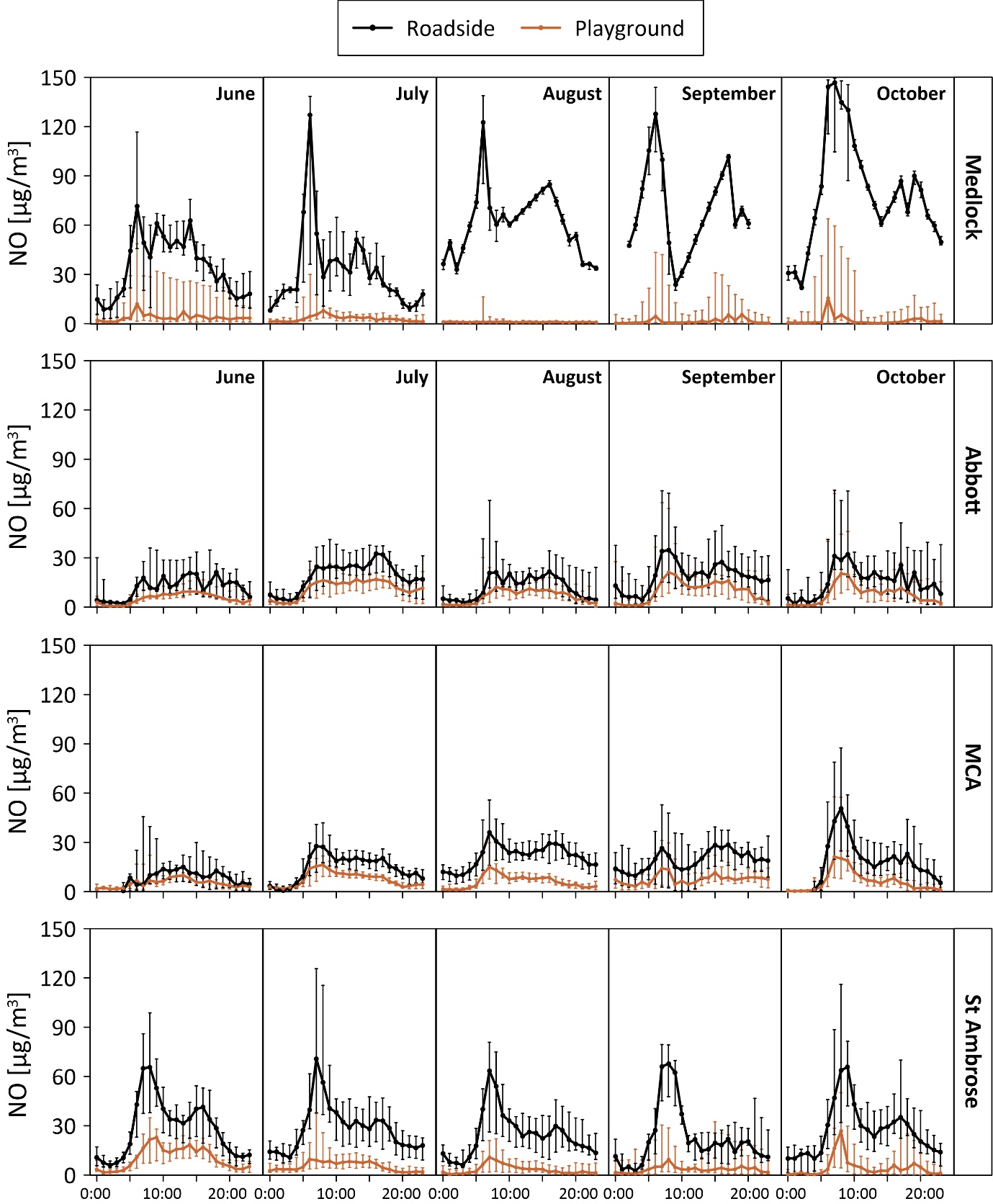


**Figure S11.** Diurnal trends of nitrogen monoxide (NO) at the roadside and at distal playground
(14 −17 m from roadside for Medlock, Abbott and St Ambrose, 7 m for MCA). Medlock, Abbott, MCA and St Ambrose.


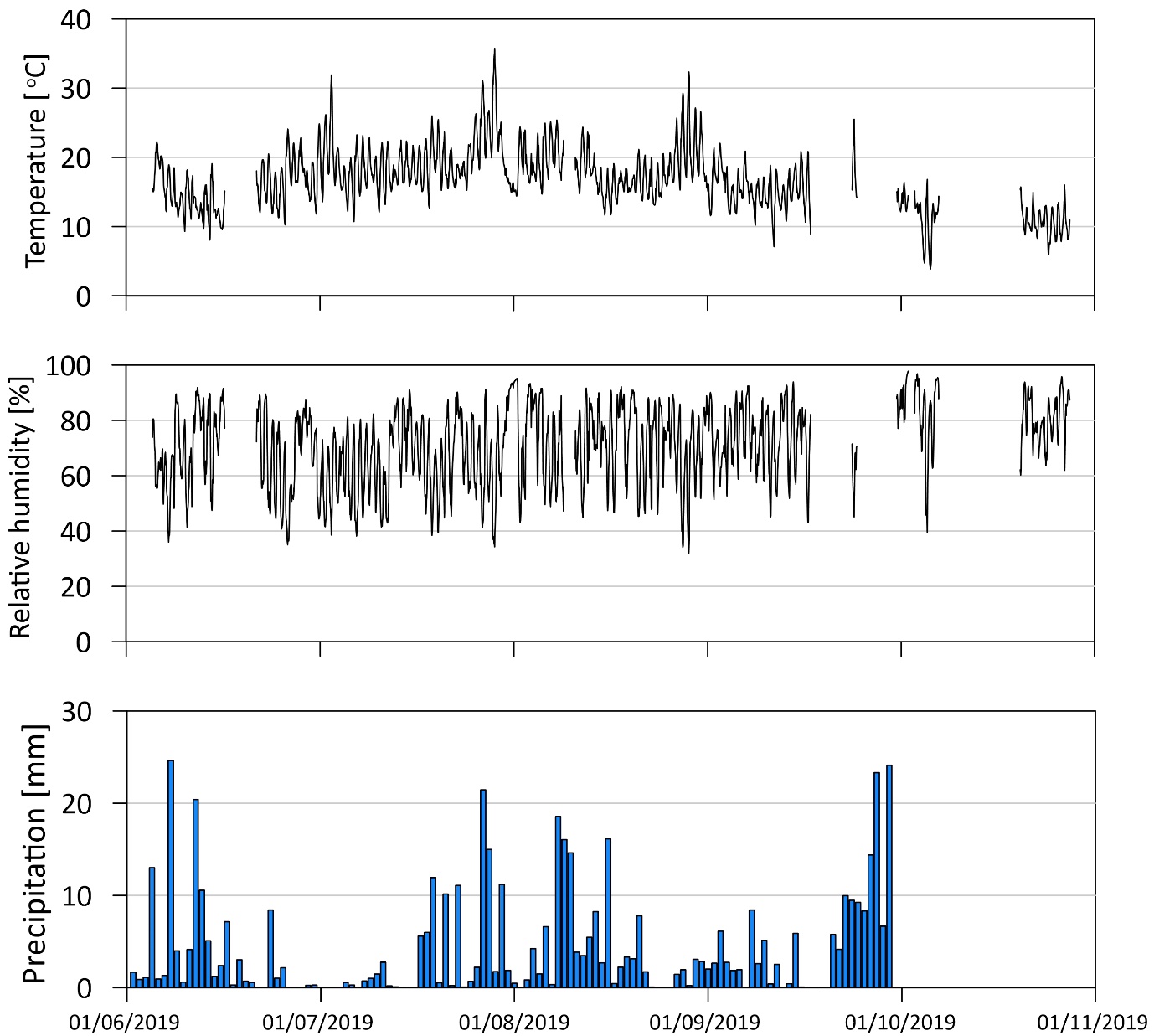


**Figure S12.** Temperature and relative humidity measured at the roadside and at distal playground (14 −17 m from roadside for Medlock, Abbott and St Ambrose, 7 m for MCA); and precipitation for NW England).


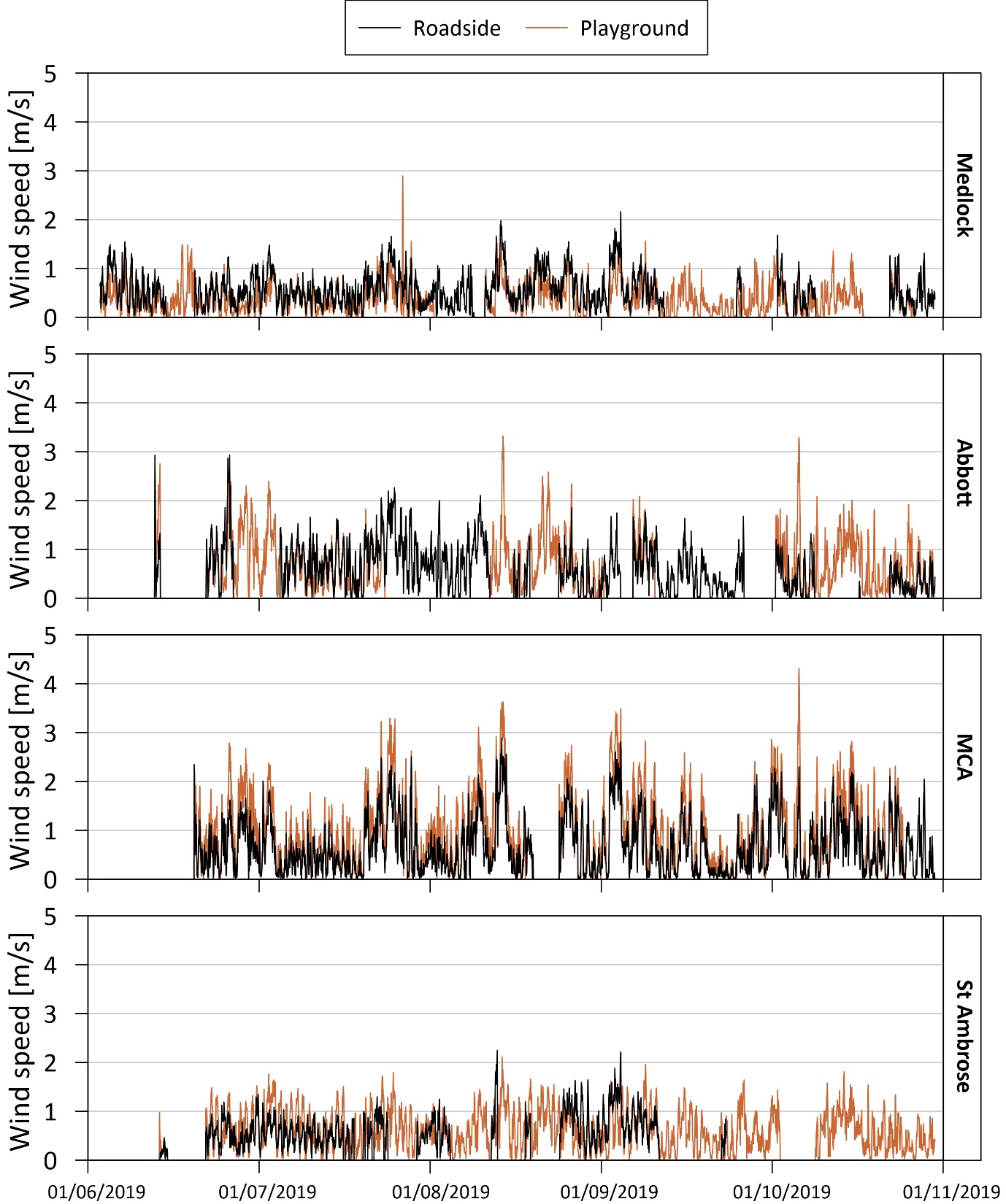


**Figure S13.** Wind speed measured at the roadside and at distal playground (14 −17 m from roadside for Medlock, Abbott and St Ambrose, 7 m for MCA). The absence of data reflects sensor(s) malfunction.

**
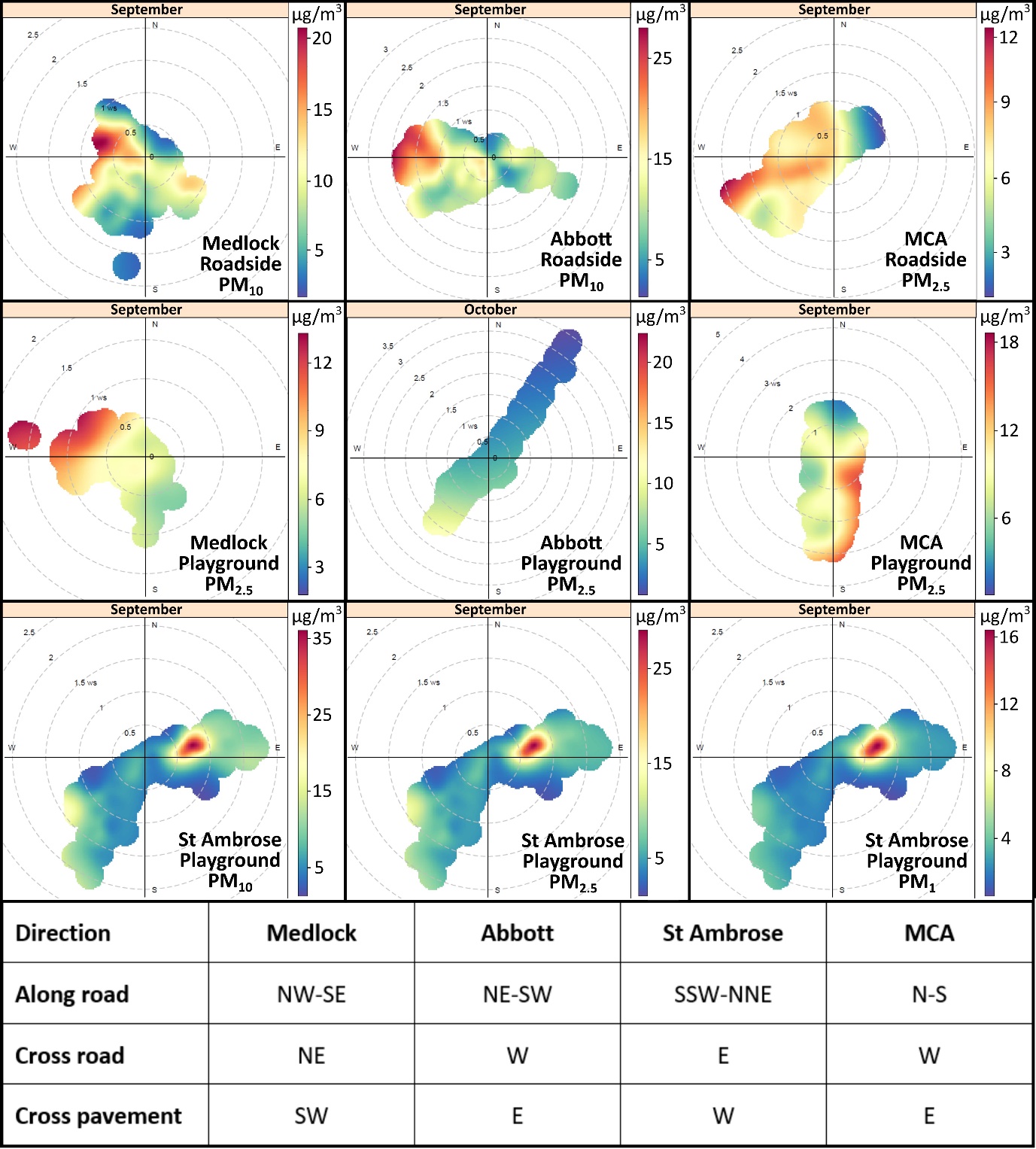
**

**Figure S14.** Selected bivariate polar plots for PM_10_, PM_2.5_ and PM_1_ at the roadside and at distal playground at Medlock, Abbott, MCA and St Ambrose.

**Table S1.** Monthly averages of PM_10_, PM_2.5_ and PM_1_ levels (Cambri)

|  | **Month** | **Medlock** | | **Abbott** | | **MCA** | | **St Ambrose** | |
| --- | --- | --- | --- | --- | --- | --- | --- | --- | --- |
|  |  | **Roadside** | **Playground** | **Roadside** | **Playground** | **Roadside** | **Playground** | **Roadside** | **Playground** |
| PM10 | June | 9.7 | 8.3 | 8.0 | 8.6 | - | 10.3 | - | 5.6 |
|  | July | 8.4 | 8.2 | 9.5 | 7.8 | 6.1 | 9.3 | 6.3 | - |
|  | August | 9.4 | 8.6 | 11.2 | 8.8 | 7.9 | 10.4 | 9.2 | 6.7 |
|  | September | 9.9 | 10.4 | 12.9 | 9.8 | 10.2 | 13.1 | 8.8 | 8.9 |
|  | October | 6.5 | 7.9 | - | - | 7.7 | - | 8.8 | 7.8 |
| PM2.5 | June | 6.6 | 6.0 | 5.0 | 6.6 | - | 7.7 | - | 4.4 |
|  | July | 5.5 | 5.6 | 5.3 | 5.3 | 4.7 | 6.2 | 4.3 | - |
|  | August | 7.2 | 6.4 | 7.0 | 6.6 | 6.1 | 7.7 | 6.9 | 6.1 |
|  | September | 7.2 | 7.7 | 8.1 | 7.4 | 7.9 | 9.6 | 6.6 | 7.4 |
|  | October | 4.9 | 5.9 | - | - | 6.0 | - | 6.6 | 6.1 |
| PM1 | June | 3.5 | 3.3 | 2.3 | 4.1 | - | 4.7 | - | 1.9 |
|  | July | 2.6 | 2.6 | 2.6 | 2.6 | 2.6 | 2.9 | 2.2 | - |
|  | August | 3.9 | 3.2 | 3.9 | 3.6 | 3.1 | 4.2 | 3.7 | 3.9 |
|  | September | 2.8 | 3.4 | 3.5 | 3.3 | 3.6 | 4.2 | 2.7 | 3.8 |
|  | October | 2.5 | 2.6 | - | - | 3.0 | - | 3.0 | 2.9 |

**Table S2.** Playground PM reductions and between-sensor measurement uncertainties; Cambri

|  | | | | **June - July** | | | | **August - October** | | | |
| --- | --- | --- | --- | --- | --- | --- | --- | --- | --- | --- | --- |
|  |  |  |  | **Medlock** | **Abbott** | **MCA** | **St Ambrose** | **Medlock** | **Abbott** | **MCA** | **St Ambrose** |
| PM_10_ | Median  [µg/m^3^] | Roadside | | 7.1 | 7.6 | 5.2 | 8.6 | 8.0 | 10.2 | 7.6 | 7.6 |
|  |  | Playground | | 6.8 | 6.2 | 7.9 | 7.6 | 7.4 | 7.9 | 9.7 | 5.1 |
|  | PM reduction | | | 5% | 18% | -52% | 12% | 7% | 23% | -28% | 32% |
|  | Between sensor uncertainty | | | 31% | 29% | 22% | 24% | 31% | 29% | 22% | 24% |
| PM_2.5_ | Median  [µg/m^3^] | Roadside | | 5.1 | 4.6 | 4.1 | 6.6 | 5.6 | 6.3 | 5.9 | 5.8 |
|  |  | Playground | | 4.9 | 4.4 | 5.3 | 5.5 | 5.6 | 5.7 | 7.0 | 4.7 |
|  | PM reduction | | | 2% | 6% | -30% | 17% | 0% | 10% | -19% | 19% |
|  | Between sensor uncertainty | | | 12% | 12% | 15% | 10% | 12% | 12% | 15% | 10% |
| PM_1_ | Median  [µg/m^3^] | | Roadside | 2.2 | 2.2 | 2.2 | 2.4 | 2.4 | 3.0 | 2.8 | 2.7 |
|  |  |  | Playground | 2.3 | 2.1 | 2.7 | 2.1 | 2.7 | 2.8 | 3.1 | 2.4 |
|  | PM reduction | | | 0% | 2% | -21% | 16% | -12% | 6% | -12% | 11% |
|  | Between sensor uncertainty | | | 13% | 9% | 14% | 11% | 13% | 9% | 14% | 11% |

**Table S3.** Playground PM reductions and between-sensor measurement uncertainties; POPS & BC

|  | | | **Abbott** | **MCA** | **St Ambrose** |
| --- | --- | --- | --- | --- | --- |
| **PM_2.5_** | **Median value [µg/m^3^]** | Roadside | 5.52 | 3.60 | 4.86 |
|  |  | Behind tredge | 4.42 | 3.34 | 4.23 |
|  |  | Playground | 4.12 | 3.26 | 3.76 |
|  | **Median PM_2.5_ reduction** | Behind tredge | 20% | 7% | 13% |
|  |  | Playground | 25% | 9% | 23% |
|  | **Spike PM_2.5_ reduction*** | Behind tredge | 55% | 30% | 23% |
|  |  | Playground | 69% | 28% | 82% |
|  | **Between sensor uncertainty** | Behind tredge | 1% | 6% | 4% |
|  |  | Playground | 2% | 6% | 2% |
| **PM_1_** | **Median value [µg/m^3^]** | Roadside | 1.90 | 1.53 | 2.36 |
|  |  | Behind tredge | 1.56 | 1.48 | 2.17 |
|  |  | Playground | 1.43 | 1.45 | 2.06 |
|  | **Median PM_1_ reduction** | Behind tredge | 18% | 4% | 8% |
|  |  | Playground | 25% | 6% | 13% |
|  | **Spike PM_1_ reduction*** | Behind tredge | 37% | 37% | 37% |
|  |  | Playground | 44% | 56% | 70% |
|  | **Between sensor uncertainty** | Behind tredge | 1% | 3% | 2% |
|  |  | Playground | 2% | 3% | 1% |
| **PNC** | **Median value  [#/cm^3^]** | Roadside | 5545 | 5234 | 9877 |
|  |  | Behind tredge | 5111 | 4827 | 9472 |
|  |  | Playground | 4753 | 4672 | 8299 |
|  | **Median PNC reduction** | Behind tredge | 8% | 8% | 4% |
|  |  | Playground | 14% | 11% | 16% |
|  | **Spike PNC reduction*** | Behind tredge | 28% | 48% | 28% |
|  |  | Playground | 34% | 49% | 67% |
|  | **Between sensor uncertainty** | Behind tredge | 4% | 3% | 3% |
|  |  | Playground | 4% | 3% | 4% |
| **BC** | **Median value  [µg/m^3^]** | Roadside | 0.69 | 0.76 | 3.56 |
|  |  | Behind tredge | 0.74 | 0.93 | 2.55 |
|  |  | Playground | 0.72 | 0.63 | 1.81 |
|  | **Median BC reduction** | Behind tredge | -8% | -23% | 28% |
|  |  | Playground | -5% | 16% | 49% |
|  | **Spike BC reduction*** | Behind tredge | 7% | -98% | 53% |
|  |  | Playground | 41% | 22% | 100% |
|  | **Between sensor uncertainty** | Behind tredge | 10% | 5% | 7% |
|  |  | Playground | 6% | 2% | 12% |

*Spike threshold defined as 3^rd^ quartile Q3 (75^th^ percentile).

**Table S4.** Magnetic parameters for leaves

| **School** | | **Abbott** | **MCA** | **MCA** | **St Ambrose** | **St Ambrose** |
| --- | --- | --- | --- | --- | --- | --- |
| **Species** | | Ivy | WR cedar | Juniper | WR cedar | WR cedar |
| **Collection date** | | 11/11/2020 | 11/11/2020 | 11/11/2020 | 10/11/2020 | 23/04/2021 |
| **SIRM  [10^-6^ A]** | Roadside | 18 | 147 | 41 | 98 | 159 |
|  | Playground | 20 | 44 | 44 | 20 | 91 |
| **Χ_ARM_ [10^-9^ m]** | Roadside | 4 | 28 | 7 | 17 | 34 |
|  | Playground | 4 | 8 | 8 | 4 | 19 |
| **Χ_ARM_/SIRM [10^-5^ m/A]** | Roadside | 19.2 | 19.2 | 16.8 | 17.3 | 21.6 |
|  | Playground | 21.2 | 17.8 | 18.8 | 20.3 | 21.2 |
| **Estimated PM_10_ mass deposited on leaves [µg/cm^2^]** | | 39 | 201 | 89 | 124 | 263 |

**Table S5.** Estimation of % removal of local, traffic-derived PM_10_ from leaf magnetic PM loading

|  | | |
| --- | --- | --- |
| Leaf collection date | 11/11/2020 | |
| Last rainfall (>5 mm) before the collection date* | 09/11/2020 | |
| Traffic count** [vehicle/day] | 80620 | |
| Emission mass flow [mg/s]  (Litschke & Kuttler, 2008) | 9.33 | |
| Cumulative traffic-emitted PM_10_ mass [g] | 2419 | |
|  | | |
| Leaf surface area [m^2^/tree] | 2.5 | |
| SIRM for leaves [10^-6^ A] | 58.9 | |
| SIRM for synthetic magnetite [Am^2^/kg] (Maher, 1988) | 5.0 | |
| Magnetite mass concentration in roadside PM [wt.%] (Gonet et al., 2021) | 0.32 – 0.95 | |
|  | 75% PM removal  with prior rain | 50% PM removal  with prior rain |
| Magnetite mass deposited on leaves [mg/m^2^] | 8.8 | 5.9 |
| Magnetite mass deposited on a tree [mg/tree] | 22.1 | 14.7 |
| PM mass deposited on a tree [g/tree] | 2.3 – 6.9 | 1.6 – 4.6 |
| PM mass deposited on the whole ‘tredge’ (102 trees), estimated based on magnetic measurements [g] | 237 – 705 | 158 – 470 |
| **Proportion of local, traffic-derived PM_10_ deposited on the tredge [%]** | **10 − 29** | **7 – 19** |

*Meteorological data from <https://www.worldweatheronline.com> for Greater Manchester; **traffic data from 2019 (Manchester City Council).
